# A universal DNA mini-barcode for biodiversity analysis

**DOI:** 10.1186/1471-2164-9-214

**Published:** 2008-05-12

**Authors:** Isabelle Meusnier, Gregory AC Singer, Jean-François Landry, Donal A Hickey, Paul DN Hebert, Mehrdad Hajibabaei

**Affiliations:** 1 Biodiversity Institute of Ontario, University of Guelph, Guelph, Ontario, N1G 2W1, Canada; 2 Human Cancer Genetics Program, The Ohio State University, Columbus OH 43210, USA; 3 Research Centre, Agriculture and Agri-Food Canada, Ottawa, Ontario K1A 0C6, Canada; 4 Department of Biology, Concordia University, 7141 Sherbrooke Street, Montreal, Quebec H4B 1R6, Canada

## Abstract

**Background:**

The goal of DNA barcoding is to develop a species-specific sequence library for all eukaryotes. A 650 bp fragment of the cytochrome *c *oxidase 1 (CO1) gene has been used successfully for species-level identification in several animal groups. It may be difficult in practice, however, to retrieve a 650 bp fragment from archival specimens, (because of DNA degradation) or from environmental samples (where universal primers are needed).

**Results:**

We used a bioinformatics analysis using all CO1 barcode sequences from GenBank and calculated the probability of having species-specific barcodes for varied size fragments. This analysis established the potential of much smaller fragments, mini-barcodes, for identifying unknown specimens. We then developed a universal primer set for the amplification of mini-barcodes. We further successfully tested the utility of this primer set on a comprehensive set of taxa from all major eukaryotic groups as well as archival specimens.

**Conclusion:**

In this study we address the important issue of minimum amount of sequence information required for identifying species in DNA barcoding. We establish a novel approach based on a much shorter barcode sequence and demonstrate its effectiveness in archival specimens. This approach will significantly broaden the application of DNA barcoding in biodiversity studies.

## Background

DNA barcoding seeks to develop a comprehensive species-specific sequence library for all eukaryotes [1]. The 650 bp mitochondrial cytochrome *c *oxidase 1 (CO1, *cox1*) DNA barcode [2] is easily sequenced and provides greater than 97% species-level specificity for birds [3], mammals [4], fishes [5], and various arthropods [6]. However, conventional DNA barcoding encounters two problems. First, DNA degradation in archival specimens and processed biological material (i.e. food products) often prevents the recovery of PCR fragments longer than 200 bp, impeding barcode recovery [7-9]. Second, current approaches cannot be used for comprehensive analysis of environmental samples because high sequence variability necessitates the use of distinct primer sets for each major taxonomic group. In this study, we propose the use of a "mini-barcode" sequence to overcome these problems. We begin by identifying the minimum amount of sequence information required for accurate species identification. We then test the gain in amplification success for smaller fragments in specimens with degraded DNA. Finally, by targeting conserved priming sites within the barcode region we develop primers with the universality required for the analysis of all major eukaryotes.

## Results and Discussion

**Figure 1:**
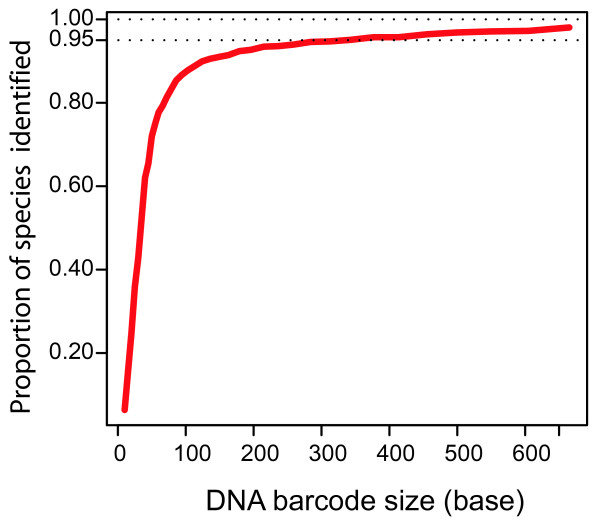
**Short DNA barcode sequences (less than 150 bp) provide efficient taxonomic sequence tags**. Increase in species resolution with increasing sequence length of cytochrome c oxidase 1.

To determine how much sequence information is required for identifications, we retrieved all CO1 barcode sequences from GenBank and calculated the probability of having species-specific barcodes for varied size fragments (Figure 1). Our analysis shows that while full-length DNA barcodes perform best (97% species resolution), 90% identification success is obtained with 100 bp regions and 95% success with 250 bp barcodes. In another words, in 90% of the species tested a DNA barcode of only 100 bp contains nucleotide substitution(s) specific to members of a particular species. Having established the potential of mini-barcodes to identify unknown specimens, we proceeded to design primers and test their performance.

**Figure 2:**
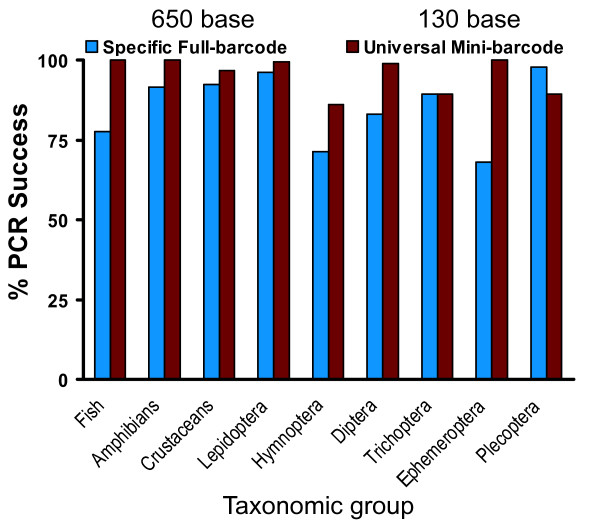
**Amplification success for a 130 bp amplicon of CO1 versus the standard 650 bp**. The mini-barcodes were amplified with a single primer set whereas the full-length DNA barcode was amplified using different, taxon-specific primers.

We designed universal primers for amplifying mini-barcodes by aligning CO1 sequences from all major eukaryote groups (animals, fungi, plants, and protists) and identifying conserved amino acid strings for primers in the size range 120–150 bp. We selected a single primer set for a 130 bp amplicon (see below). We tested the ability of this universal primer set to amplify DNA extracts from 1,566 specimens derived from 691 species of mammals, fishes, birds, and insects (mainly Lepidoptera, Hymenoptera, Ephemeroptera, Plecoptera, and Trichoptera). Moreover, we examined another 330 DNA extracts from plants, fungi, and macroalgae (Additional file 1). We obtained PCR amplicons from 92% of these species (Additional file 2) and compared this success to that obtained via routine barcoding. We were able to amplify mini-barcodes with greater success than full-length sequences in all groups but one (9% lower success in Plecoptera) (Figure 2). We verified that the 130 bp amplicons were CO1 by sequencing approximately half of them (results not shown).

**Figure 3:**
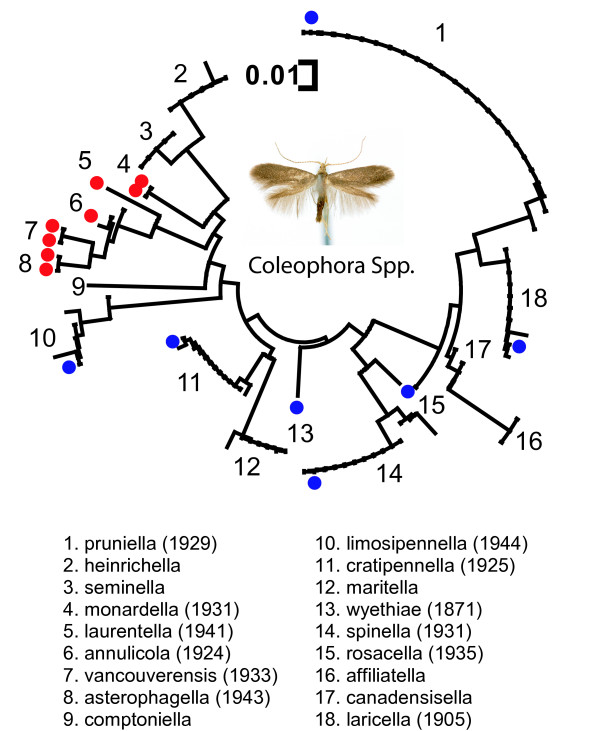
**The 130 bp amplicon is reliably amplified from old museum specimens**. Example here is from the species of the Lepidoptera genus *Coleophora*. The tree is reconstructed using neighbor-joining method [12] with Kimura two-parameter evolutionary distances [13]. Museum samples are shown by blue and red (for type specimens) dots. Collection dates are shown in parentheses.

One important application of mini-barcodes lies in obtaining sequence information from old type specimens. We tested this approach on a collection of *Coleophora *(Order Lepidoptera) which are difficult to identify because of their small size and cryptic morphology. We successfully sequenced all 15 dried museum specimens collected from 1871 to 1944 (including 7 type specimens) in one PCR pass with the universal mini-barcode primers and compared these specimens to 84 full-length barcodes from recently collected specimens of this genus (Figure 3). Comparison of sequence information obtained from these old specimens with recently collected samples provided excellent corroboration and species-level resolution. Mini-barcode sequences from old museum specimens formed monophyletic groups, containing zero or very low sequence divergence, with freshly collected specimens of the corresponding species (Figure 3).

## Conclusion

The mini-barcode system dramatically broadens the applications of DNA barcoding. We have now demonstrated that sequence information can be reliably obtained from archival specimens or those with degraded DNA. Further, the universality of the primers enables the recovery of comprehensive barcode information from environmental mixtures. Finally, the short universally-primed amplicon is ideal for sequence characterization through new parallelized high-throughput sequencing technologies, allowing inexpensive but comprehensive studies of biodiversity to be a realistic goal.

## Methods

### Bioinformatics analysis

Metazoan COI sequences bearing the "BARCODE" keyword were downloaded from GenBank using the NCBI eFetch tool. Barcodes that were less than 650 bases in length were eliminated, leaving a dataset of 6,695 barcode sequences from 1,587 species. For various sizes of 5'-end minibarcodes, ranging in size from 10 bases up to the full-length of the barcode sequence, we analyzed the number of species that could be uniquely identified (to the exclusion of other species) using that sequence.

### Specimens and their taxonomic coverage

All DNA extracts were obtained from different barcoding projects in the Canadian Centre for DNA Barcoding and external collaborators. We selected these samples considering maximum taxonomic range.

### Primer design strategy

We selected the 5' end of the barcode region targeting a 100–150 base amplicon. By comparing a wide range of taxa in this region, we found well-conserved strings of amino acids across all taxa in priming sites. Interestingly, this high level of conservation is also evident at nucleotide level. We designed multiple oligos by using the Primer3 program [10] and considering physical and structural properties of oligos (such as annealing temperature, G+C percentage, and self-complementarity). We selected the primer Uni-MinibarR1: 5'-GAAAATCATAATGAAGGCATGAGC-3' for further testing as it represented the highest similarity – especially at the 3' end – to other taxa. A similar strategy was used for designing a forward primer: Uni-MinibarF1: 5'-TCCACTAATCACAARGATATTGGTAC-3'. This primer is positioned in the same region as other common barcoding primers are located. We attached M13 forward and reverse tails to our forward and reverse primers, respectively, to facilitate the high throughput sequencing process. These tails did not decrease the PCR success.

### PCR Optimization Strategy

PCR reactions were performed using a standard PCR pre-mix [11]. We used the above mentioned universal primer set in all the reactions in a touch up PCR program: 95°C for 2 min, followed by 5 cycles of 95°C-1 min, 46°C-1 min, and 72°C-30 sec, followed by 35 cycles of 95°C-1 min, 53°C-1 min, and 72°C-30 sec, and finally a final extension at 72°C for 5 min. We used a  Mastercycler ep gradient S (Eppendorf, Mississauga, ON, Canada) thermalcycler. We included two negative control reactions (no DNA template) in all our PCR 96-well plates. To compare the universal mini-barcode primer set with the specific full-length primers we amplified DNA extracts using taxonomically specific primer sets (i.e. 2 primer sets for fish species) [11].

### PCR amplification verification and sequencing

PCR products were visualized on a 2% E-gel^® ^96 Agarose (Invitrogen, Burlington, ON, Canada). The bands on E-gel were used as a measure of PCR success. To verify the amplification of the target region, we sequenced 747 PCR products from at least 363 species. Standard BigDye kits (Applied Biosystems, Foster City, CA) were used for sequencing. Sequencing reactions were cleaned up by using Agencourt^® ^CleanSEQ^® ^kit (Agencourt Bioscience Corporation, Beverly, MA). The sequences were run bidirectionally on a 3730xl DNA analyzer (Applied Biosystems, Foster City, CA, USA), edited with Sequencher™ (Gene Codes Corporation, Ann Arbor, MI), and aligned using BioEdit version 7.0.5.3.

## Authors' contributions

IM performed molecular methods, assisted in project design and data analysis, and edited the manuscript. GACS gathered sequence information from GenBank, designed and conducted bioinformatics analysis, and edited the manuscript. J–FL carried out taxonomic analysis and edited the manuscript. DAH aided the study design, provided tools/reagents, and edited the manuscript. PDNH aided the study design, provided tools/reagents, and edited the manuscript. MH designed the project, performed DNA barcode data analysis, and wrote the manuscript. All authors read and approved the final manuscript.

## Supplementary Material

Additional file 1 Specimens used in this study. Click here for file

Additional file 2 Mini-barcodes of 130 bp can be PCR amplified from the majority of specimens using a single universal primer set for all major eukaryotic lineages. Click here for file

## References

[B1] Marshall E (2005). Taxonomy. Will DNA bar codes breathe life into classification?. Science.

[B2] Hebert PD, Cywinska A, Ball SL, deWaard JR (2003). Biological identifications through DNA barcodes. Proc Biol Sci.

[B3] Hebert PD, Stoeckle MY, Zemlak TS, Francis CM (2004). Identification of birds through DNA barcodes. PLoS Biology.

[B4] Hajibabaei M, Singer GA, Clare EL, Hebert PDN (2007). Design and applicability of DNA arrays and DNA barcodes in biodiversity monitoring. BMC Biol.

[B5] Ward RD, Zemlak TS, Innes BH, Last PR, Hebert PD (2005). DNA barcoding Australia's fish species. Philos Trans R Soc Lond B Biol Sci.

[B6] Hajibabaei M, Janzen DH, Burns JM, Hallwachs W, Hebert PDN (2006). DNA barcodes distinguish species of tropical Lepidoptera. Proceedings of the National Academy of Sciences of the United States of America.

[B7] Goldstein PZ, Desalle R (2003). Calibrating phylogenetic species formation in a threatened insect using DNA from historical specimens. Molecular Ecology.

[B8] Hajibabaei M, Smith MA, Janzen DH, Rodriguez JJ, Whitfield JB, Hebert PDN (2006). A minimalist barcode can identify a specimen whose DNA is degraded. Molecular Ecology Notes.

[B9] Wandeler P, Hoeck PE, Keller LF (2007). Back to the future: museum specimens in population genetics. Trends Ecol Evol.

[B10] Rozen S, Skaletsky H (2000). Primer3 on the WWW for general users and for biologist programmers. Methods Mol Biol.

[B11] The Canadian Centre for DNA Barcoding. http://www.dnabarcoding.ca/pa/ge/research/protocols/amplification.

[B12] Saitou N, Nei M (1987). The neighbor-joining method: a new method for reconstructing phylogenetic trees. Mol Biol Evol.

[B13] Kimura M (1980). A simple method for estimating evolutionary rates of base substitutions through comparative studies of nucleotide sequences. Journal of molecular evolution.

