# Supplementary material for: A universal DNA mini-barcode for biodiversity analysis
Source: BMC Genomics. 2008 May 12;9:214. doi: 10.1186/1471-2164-9-214 (PMC2396642; doi:10.1186/1471-2164-9-214)
Supplement: Additional file 1 — Specimens used in this study. [file 1471-2164-9-214-S1.pdf]

Additional file 1. Specimens used in this study

Taxonomic group		N	Identification	Process ID
**Fungi**	Agaricus	38	*Agaricus arvensis*	WC-145
			Agaricus arvensis	WC-261
			Agaricus arvensis	WC-280
			Agaricus arvensis	WC-344
			Agaricus arvensis	WC-345
			Agaricus arvensis	WC-346
			Agaricus arvensis	WC-847
			Agaricus arvensis	WC-848
			Agaricus augustus	WC-19
			Agaricus augustus	WC-76
			Agaricus augustus	WC-106
			Agaricus augustus	WC-129
			Agaricus augustus	WC-133
			Agaricus augustus	WC-138
			Agaricus augustus	WC-209
			Agaricus augustus	WC-210
			Agaricus augustus	WC-211
			Agaricus augustus	WC-212
			Agaricus augustus	WC-213
			Agaricus augustus	WC-253
			Agaricus bisporus	MC-310
			Agaricus bisporus	MC-324
			Agaricus bisporus	MC-441
			Agaricus bisporus	MC-450
			Agaricus bisporus	MC-452
			Agaraicus campestris	WC-83
			Agaraicus campestris	WC-413.1
			Agaraicus campestris	WC-418
			Agaraicus campestris	MW-5
			Agaraicus campestris	MW-6
			Agaraicus campestris	MW-7
			Agaraicus campestris	MW-8
			Agaraicus campestris	MW-10
			Agaricus subfloccosus	WC-781
			Agaricus subfloccosus	WC-721
			Agaricus subfloccosus	WC-722
			Agaricus subfloccosus	WC-725
			Agaricus subfloccosus	WC-729
	AMF spore	8	*Glomeromycota*	CL151
			Gigaspora gigantea	NC110A12
			Glomeromycota	LTR1R53
			Glomeromycota	UK-126
			Gigaspora rosea	BR151A-1
			Entrophospora colombiana	CL356-1
			Glomus etunicatum	NB119-1
			Scutellospora coralloides	CA260-11
	AMF concentrated in roo	94	*Glomus eburneum*	MX917-6
			Scutellospora calospora	AU222-16
			Acaulospora laevis	AU211-11
			Archaeospora leptoticha	JA401A-5

			Acaulospora tuberculata	VZ103E-16
			Entrophospora colombiana	GA101-1
			Gigaspora decipiens	AU102-2
			Glomus mosseae	HO102-8
			Entrophospora contigua Orange	WV201-11
			Archaeospora trappei	AK102A-7
			Glomus eburneum	AZ414A-7
			Glomus clarum	AU404-1
			Scutellospora verrucosa	HA150A-12
			Gigaspora margarita	WV205A-11
			Paraglomus occultum	HA771-8
			Archaeospora leptotichum	CR31-4
			Acaulospora sp.	FL736-2
			Acaulospora foveata	CR315-7
			Glomus claroideum	DN987-12
			Glomus patchy	AZ151A-3
			Glomus mosseae	CU114-7
			Scutellospora castanea	BEG1-2
			Glomus geosporum	CI102
			Acaulospora delicata	CI103
			Glomus versiforme	BEG47-5
			Glomus manihot	KY305-1
			Acaulospora delicata	NI117-4
			Glomeromycota	CA260-11
			Glomus mosseae	IA190-4
			Scutellospora persica	MA461B-5
			Scutellospora dipurpurascens	IL101-9
			Glomus intraradices	IC101-10
			Gigaspora albida	CL151-6
			Glomus trimurales	BR608-9
			Entrophospora infrequens	IN215-4
			Glomus deserticola	NC302A-9
			Gigaspora margarita	JA201A-1
			Glomus intraradices	IS185-8a
			Glomeromycota	IS185-8b
			Glomus versiforme	IT104-4
			Glomus sp.	NV105-9
			Acaulospora scrobiculata	CU130-4
			Acaulospora morrowiae	FL219B-1
			Paraglomus occultum	VZ103C-6
			Acaulospora lacunosa	NH102-6
			Entrophospora colombiana	NB104C-6
			Scutellospora heterogama	WV858B-1
			Glomus sinuosum	MD126-7
			Scutellospora heterogama	SN722-1
			Scutellospora fulgida	NC303A-9
			Acaulospora morrowiae	BR225-6
			Entrophospora kentinensis	TW111A-3
			Glomus sp.	TW117-0
			Glomus intraradices	TU101-1
			Acaulospora mellea	CL697-9
			Glomus mosseae	NB114-9

			Acaulospora morrowiae	CR201-7
			Glomus luteum	ON201A-3
			Scutellospora gregaria	NC210-1
			Glomus eburneum	UK121-9
			Paraglomus occultum	OR924-8
			Glomus etunicatum	SP108E-8
			Scutellospora calospora	NC128-9
			Glomus gold	KE102-B
			Acaulospora morrowiae	KR102-6
			Paraglomus brasilianum	BR105-8
			Paraglomus occultum	CR102-7
			Glomus mosseae	UK126-8
			Glomus aggregatum	FL3120-10
			Glomus caledonium	HA692B-7
			Glomus caledonium	UK112A-7
			Acaulospora trappei	SF113-6
			Archaeospora gerdemannii	MT106-6
			Glomus halonatum	KS101-6
			Acaulospora morrowiae	EY106-6
			Glomus etunicatum YV	FL312C-9
			Glomeromycota	FL327B-9
			Glomus Yellow	PA116A-7
			Scutellospora pellucida	CL750A-9
			Glomus luteum?	IN101A-1
			Glomus monosporum	WY107-7
			Archaeospora trappei	MG110-5
			Scutellospora erythropa	HA150-B
			Glomus creamy white	IA171-2
			Glomeromycota	NC110A-12
			Glomus hyalinulum?	NC268A-6
			Archaeospora leptoticha	ON205-1
			Scutellospora erythropa	RI275B-13
			Glomus monosporum	IT102-5
			Glomus spurcum	SC151-6
			Glomus leptotichum	SC704-6
			Glomus etunicatum	MX116A-9
			Glomus fragilistratum	DN988-10
			Glomus viscosum	MD216-6
	Hyphomycetes	30	Cylindrocarpon didymum	P10_10_03
			Lemonniera aquatica	WD(A)_00_2
			Dimorphospora foliicola	GMW_07_09
			Variocosporum sp	Spw_08_02
			Heliscus lugdunensis	H4_2_4
			Heliscus lugdunensis	H8_2_1
			Alatospora acunminata	Spw_fl_01_05
			Tricladium angulatum	Rh_01_6
			Cylindrocarpon sp	GWM_07_02
			Variocosporum elodeae	WD(B)_00_1
			Clavariopsis aquatica	WD(A)_00_1
			Flagellospora curvula	SHM_D_01_5
			Anguillospora longissima	Rh_01_1
			Tetracladium setigerum	Rh_01_16

			Tetracladium elegans	SHM_D_01_1
			Epiccocum nigrum	H9_06_I2
			Lemonniera aquatica	CCM_F_04480
			Alatospora acuminata	CCM_F_37194
			Tricladium angulatum	CCM _F_10200
			Clavariopsis aquatica	CCM_F_10491
			Flagellospora curvula	CCM_F_13399
			Flagellospora curvula	CCM_F_20799
			Anguillospora longuissima	CCM_F_11791
			Tetracladium setigerum	CCM_F_20987
			Lunulospora curvula	CCM_F_10503
			Filosporella versimorpha	CCM_F_10403
			Heliscella stellata	CCM_F_665
			Tetracladium marchialanum	CCM_F_19399
			Lemonniera terrestris	CCM_F_07782
			Lemonniera centrosphaera	CCM_F_21094
	ATCC samples	19	Agaricus campestris	26816 FD
			Agaricus campestris	26817 FD
			Agaricus campestris	26815 FD
			Aspergillus Oryzae	20386 FD
			Aspergillus flavus; var. oryzae	42149 FD
			Aspergillus terreus	12238 FD
			Byssochlamys fulva	36841 FD
			Aspergillus fumigatus	24913 FD
			Aspergillus tardus	58802 FD
			Aspergillus terreus	24839 FD
			Aspergillus terreus; 77 (45-2)	58095 FD
			Agaricus bisporus	10892 FZ
			Agaricus bisporus	18801 FZ
			Agaricus arvensis	22036 FZ
			Agaricus subperonatus	22040 FZ
			Agaricus subfloccosus RWK 1397	MYA-3441 FZ
			Agaricus blazei	76739 FZ
			Agaricus bisporum fungisem h26	MYA-1186 FZ
			Penicillium multicolor	24723 FZ
		4	Basidiomycota	NIV07-001
			Basidiomycota	NIV07-002
			Basidiomycota	NIV07-003
			Basidiomycota	5(1/10/06) Pedro
			Basidiomycota	8(1/10/06) Pedroc
	FASEL (Ontario flora)	94	Basidiomycota	153A06
			Basidiomycota	151A06
			Basidiomycota	147B06
			Basidiomycota	149B06
			Basidiomycota	153C06
			Basidiomycota	162A06
			Basidiomycota	1LEC06
			Basidiomycota	2LEC06
			Basidiomycota	152A06
			Basidiomycota	148B06
			Basidiomycota	149A06
			Basidiomycota	157A06

			Basidiomycota	152B06
			Basidiomycota	168A06
			Basidiomycota	162C06
			Basidiomycota	148A06
			Basidiomycota	162B06
			Basidiomycota	165A06
			Basidiomycota	167B06
			Basidiomycota	299A06
			Basidiomycota	170A06
			Basidiomycota	295LEC06
			Basidiomycota	300A06
			Basidiomycota	172B06
			Basidiomycota	172A06
			Basidiomycota	164C06
			Basidiomycota	H54DA06
			Basidiomycota	H51DA06
			Basidiomycota	296C06
			Basidiomycota	292D06
			Basidiomycota	292E06
			Basidiomycota	292C06
			Basidiomycota	296A06
			Basidiomycota	231B06
			Basidiomycota	238A06
			Basidiomycota	232A06
			Basidiomycota	233B06
			Basidiomycota	239B06
			Basidiomycota	223A06
			Basidiomycota	239A06
			Basidiomycota	262A06
			Basidiomycota	266A06
			Basidiomycota	246B2906
			Basidiomycota	258A06
			Basidiomycota	262B06
			Basidiomycota	3B206
			Basidiomycota	126B206
			Basidiomycota	183A206
			Basidiomycota	190D206
			Basidiomycota	69A206
			Basidiomycota	248B05
			Basidiomycota	270A05
			Basidiomycota	281A05
			Basidiomycota	75B05
			Basidiomycota	199C05
			Basidiomycota	199A05
			Basidiomycota	81C05
			Basidiomycota	81B05
			Basidiomycota	81A05
			Basidiomycota	85A05
			Basidiomycota	92A05
			Basidiomycota	92A205
			Basidiomycota	92FBA05
			Basidiomycota	92B05

			Basidiomycota	64A05
			Basidiomycota	65A05
			Basidiomycota	66A05
			Basidiomycota	67A05
			Basidiomycota	69A05
			Basidiomycota	70A05
			Basidiomycota	75A05
			Basidiomycota	61A05
			Basidiomycota	62A05
			Basidiomycota	63A05
			Basidiomycota	68A05
			Basidiomycota	59C05
			Basidiomycota	59B05
			Basidiomycota	57A05
			Basidiomycota	53A05
			Basidiomycota	49B05
			Basidiomycota	49A05
			Basidiomycota	47A05
			Basidiomycota	39B05
			Basidiomycota	39A05
			Basidiomycota	39A205
			Basidiomycota	41A05
			Basidiomycota	41B05
			Basidiomycota	42A05
			Basidiomycota	43A05
			Basidiomycota	44A05
			Basidiomycota	46A05
			Basidiomycota	37A05
			Basidiomycota	31A05
			Basidiomycota	21A05
**Protists**	Red algae	6	Callophyllis edentata	GWS004167
			Ahnfeltia plicata	GWS003520
			Nemalion helminthoides 2	GWS004031
			Corallina officinalis 2	GWS007213
			Bangia fuscopurporea	GWS006061
			Schizymenia pacifica	GWS004854
	Brown algae	6	Macrocystis integrifolia	GWS002852
			Analipus japonicus	GWS004078
			Elachista sp.	GWS005237
			Chordaria flagelliformis	GWS005281
			Laminaria digitata	GWS005663
			Desmarestia aculeata	GWS005886
	Green algae	6	Acrosiphonia sp.	GWS007351
			Enteromorpha sp.	GWS007920
			Monostroma sp.	GWS007972
			Codium setchellii	GWS002933
			Cladophora sericea	GWS003029
			Urospora penicIliformis	GWS002687
**Plants**	24		Opuntia rufida	Local
			Catlleya sp.	Local
			Portulacaria afra	Local
			Aloe zebrina	Local

			Dieffenbachia sp.	Local
			Rhododendrum brachicarpum	Local
			Rhododendrum fortunei	Local
			Asclepius syriaca	Local
			Equisetum arvense	Local
			Euphorbia esula	Local
			Impatiens capensis	Local
			Asimina triloba	Local
			Carya ovata	Local
			Acer platanoides	Local
			Vitis riparia	Local
			Quercus bicolor	Local
			Taxus canadensis	Local
			Ginkgo biloba	Local
			Pinus banksiana	Local
			Pinus armandii	Local
			Juniperus virginiana	Local
			Picea pungens	Local
			Larix decidia 'pendula'	Local
			Thuja occidentalis	Local
**Mammals**	Mammals	92	Rattus remotus	ABRVN207-06
			Aselliscus stoliczkanus	ABRVN183-06
			Leopoldamys edwardsi	ABRVN169-06
			Rhinolophus stheno	ABRVN172-06
			Rhinolophus pearsonii	ABRVN164-06
			Myotis rosseti	ABRVN305-06
			Rhinolophus pusillus	ABRVN310-06
			Rhinolophus chaseni	ABRVN300-06
			Rhinolophus shameli	ABRVN301-06
			Megaderma spasma	ABRVN303-06
			Pipistrellus ceylonicus	ABRVN293-06
			Megaerops niphanae	ABRVN215-06
			Hylopetes spadiceus	ABRVN206-06
			Eonycteris spelaea	ABRVN392-06
			Miniopterus magnater	ABRVN379-06
			Scotophilus kuhlii	ABRVN356-06
			Kerivoula papillosa	ABRVN363-06
			Kerivoula hardwickii	ABRVN349-06
			Hipposideros galeritus	ABRVN353-06
			Megaderma lyra	ABRVN330-06
			Hipposideros larvatus	ABRVN316-06
			Niviventer fulvescens	ABRVN507-06
			Maxomys moi	ABRVN511-06
			Crocidura attenuata	ABRVN499-06
			Rhinolophus affinis	ABRVN494-06
			Macroglossus sobrinus	ABRVN458-06
			Macroglossus minimus	ABRVN447-06
			Taphozous melanopogon	ABRVN433-06
			Pipistrellus tenuis	ABRVN391-06
			Rhinophylla fischerae	ABECA050-06
			Artibeus gnomus	ABECA052-06
			Phyllostomus elongatus	ABECA043-06

			Mesophylla macconnelli	ABECA046-06
			Tylonycteris robustula	ABRVN554-06
			Rhinolophus lepidus	ABRVN543-06
			Hipposideros pomona	ABRVN522-06
			Mus pahari	ABRVN513-06
			Platyrrhinus infuscus	ABECA114-06
			Artibeus planirostris	ABECA116-06
			Trachops cirrhosus	ABECA107-06
			Rhinophylla pumilio	ABECA094-06
			Macrophyllum macrophyllum	ABECA100-06
			Proechimys gularis	ABECA087-06
			Oryzomys capito	ABECA065-06
			Vampyressa pusilla	ABECA227-06
			Didelphis marsupialis	ABECA211-06
			Sturnira magna	ABECA200-06
			Neacomys spinosus	ABECA175-06
			Vampyressa bidens	ABECA165-06
			Phyllostomus hastatus	ABECA160-06
			Tonatia saurophila	ABECA154-06
			Desmodus rotundus	ABECA111-06
			Philander andersoni	ABECA965-06
			Marmosa demerarae	ABECA962-06
			Oecomys bicolor	ABECA964-06
			Platyrrhinus helleri	ABECA828-06
			Oryzomys macconnelli	ABECA832-06
			Proechimys simonsi	ABECA816-06
			Carollia castanea	ABECA309-06
			Sturnira lilium	ABECA302-06
			Lophostoma carrikeri	ABSRA416-06
			Phyllostomus discolor	ABSRA408-06
			Proechimys cuvieri	ABSRA387-06
			Marmosops parvidens	ABSRA385-06
			Proechimys guyannensis	ABSRA366-06
			Neacomys paracou	ABSRA368-06
			Pteronotus parnellii	ABGYF053-06
			Proechimys steerei	ABECA983-06
			Glossophaga soricina	ABSRA496-06
			Pteronotus personatus	ABSRA477-06
			Ametrida centurio	ABSRA471-06
			Phylloderma stenops	ABSRA473-06
			Mimon crenulatum	ABSRA465-06
			Chiroderma trinitatum	ABSRA437-06
			Sturnira tildae	ABSRA435-06
			Lonchophylla thomasi	ABSRA422-06
			Hipposideros armiger	ABRVN647-06
			Pipistrellus abramus	ABRVN064-06
			Niviventer confucianus	ABRVN021-06
			Rousettus amplexicaudatus	ABRVN007-06
			Chiroderma villosum	ABSRA529-06
			Vampyrodes caraccioli	ABSRA518-06
			Artibeus cinereus	ABSRA511-06
			Rhynchonycteris naso	ABSRA505-06

			Myotis laniger	ABRVN705-06
			Suncus murinus	ABRVN707-06
			Molossus molossus	ABECA491-06
			Cyclopes didactylus	ABECA468-06
			Rattus rattus	ABECA455-06
			Oecomys concolor	ABECA421-06
			Saccopteryx bilineata	ABECA412-06
			Oryzomys yunganus	ABECA366-06
	Bats	81	Eptesicus fuscus	BCLP002-06
			Eptesicus fuscus	BCLP003-06
			Myotis septentrionalis	BCLP004-06
			Myotis septentrionalis	BCLP005-06
			Myotis lucifugus	BCLP006-06
			Myotis lucifugus	BCLP007-06
			Myotis lucifugus	BCLP008-06
			Myotis lucifugus	BCLP009-06
			Myotis lucifugus	BCLP010-06
			Myotis lucifugus	BCLP011-06
			Eptesicus fuscus	BCLP012-06
			Eptesicus fuscus	BCLP013-06
			Myotis lucifugus	BCLP014-06
			Myotis lucifugus	BCLP015-06
			Myotis lucifugus	BCLP016-06
			Myotis lucifugus	BCLP017-06
			Myotis lucifugus	BCLP018-06
			Myotis lucifugus	BCLP019-06
			Myotis lucifugus	BCLP020-06
			Myotis lucifugus	BCLP021-06
			Myotis lucifugus	BCLP022-06
			Myotis lucifugus	BCLP023-06
			Myotis lucifugus	BCLP024-06
			Myotis lucifugus	BCLP025-06
			Myotis lucifugus	BCLP026-06
			Eptesicus fuscus	BCLP027-06
			Eptesicus fuscus	BCLP028-06
			Myotis lucifugus	BCLP029-06
			Myotis lucifugus	BCLP030-06
			Eptesicus fuscus	BCLP031-06
			Eptesicus fuscus	BCLP032-06
			Eptesicus fuscus	BCLP033-06
			Eptesicus fuscus	BCLP034-06
			Eptesicus fuscus	BCLP035-06
			Eptesicus fuscus	BCLP036-06
			Eptesicus fuscus	BCLP037-06
			Eptesicus fuscus	BCLP038-06
			Myotis lucifugus	BCLP041-06
			Myotis lucifugus	BCLP042-06
			Myotis lucifugus	BCLP039-06
			Myotis lucifugus	BCLP040-06
			Myotis lucifugus	BCLP043-06
			Myotis lucifugus	BCLP044-06
			Myotis lucifugus	BCLP045-06

			Myotis lucifugus	BCLP046-06
			Eptesicus fuscus	BCLP047-06
			Eptesicus fuscus	BCLP048-06
			Myotis lucifugus	BCLP049-06
			Myotis lucifugus	BCLP050-06
			Eptesicus fuscus	BCLP051-06
			Eptesicus fuscus	BCLP052-06
			Lasiurus borealis	BCLP053-06
			Lasiurus borealis	BCLP054-06
			Lasionycteris noctivagans	BCLP055-06
			Lasionycteris noctivagans	BCLP056-06
			Lasionycteris noctivagans	BCLP057-06
			Lasionycteris noctivagans	BCLP058-06
			Lasionycteris noctivagans	BCLP059-06
			Eptesicus fuscus	BCLP060-06
			Eptesicus fuscus	BCLP061-06
			Lasionycteris noctivagans	BCLP062-06
			Lasionycteris noctivagans	BCLP063-06
			Myotis lucifugus	BCLP064-06
			Myotis lucifugus	BCLP065-06
			Myotis lucifugus	BCLP066-06
			Myotis lucifugus	BCLP067-06
			Lasionycteris noctivagans	BCLP068-06
			Lasionycteris noctivagans	BCLP069-06
			Lasionycteris noctivagans	BCLP070-06
			Lasionycteris noctivagans	BCLP071-06
			Lasionycteris noctivagans	BCLP072-06
			Lasionycteris noctivagans	BCLP073-06
			Lasionycteris noctivagans	BCLP074-06
			Lasionycteris noctivagans	BCLP075-06
			Nycticeius humeralis	BCLP076-06
			Nycticeius humeralis	BCLP077-06
			Lasionycteris noctivagans	BCLP078-06
			Lasionycteris noctivagans	BCLP079-06
			Lasionycteris noctivagans	BCLP080-06
			Lasionycteris noctivagans	BCLP081-06
			Lasionycteris noctivagans	BCLP082-06
**Birds**		94	Ardea cocoi	KBARG008-07
			Buteogallus urubitinga	KBARG016-07
			Platalea ajaja	KBARG024-07
			Amblyramphus holosericeus	KBARG032-07
			Thraupis bonariensis	KBARG039-07
			Phalacrocorax atriceps	KBARG047-07
			Columba livia	KBARG055-07
			Hymenops perspicillatus	KBARG063-07
			Phalacrocorax brasilianus	KBARG071-07
			Serpophaga subcristata	KBARG079-07
			Progne tapera	KBARG087-07
			Tringa flavipes	KBARG007-07
			Thraupis sayaca	KBARG015-07
			Phalacrocorax atriceps	KBARG023-07
			Larus dominicanus	KBARG031-07

				KBAR160-06
			Progne chalybea	KBARG046-07
			Sterna superciliaris	KBARG054-07
			Tyrannus melancholicus	KBARG062-07
			Tringa solitaria	KBARG070-07
			Carduelis magellanica	KBARG078-07
			Phalacrocorax atriceps	KBARG086-07
			Zenaida auriculata	KBARG006-07
			Knipolegus hudsoni	KBARG014-07
			Progne chalybea	KBARG022-07
			Butorides striata	KBARG030-07
			Progne tapera	KBARG038-07
			Charadrius collaris	KBARG045-07
			Calidris melanotos	KBARG053-07
			Spheniscus magellanicus	KBARG061-07
			Patagioenas picazuro	KBARG069-07
			Buteo magnirostris	KBARG077-07
			Progne chalybea	KBARG085-07
			Caracara plancus	KBARG093-07
			Progne tapera	KBARG005-07
			Caracara plancus	KBARG013-07
			Tringa solitaria	KBARG021-07
			Spheniscus magellanicus	KBARG029-07
			Phylloscartes ventralis	KBARG037-07
			Tyrannus savana	KBARG044-07
			Myiodynastes maculatus	KBARG052-07
			Pachyramphus validus	KBARG060-07
			Ictinia plumbea	KBARG068-07
			Callonetta leucophrys	KBARG076-07
			Celeus lugubris	KBARG084-07
			Spheniscus magellanicus	KBARG092-07
			Anairetes flavirostris	KBARG004-07
			Cygnus melancoryphus	KBARG012-07
			Thlypopsis sordida	KBARG020-07
			Myiodynastes maculatus	KBARG028-07
			Caracara plancus	KBARG036-07
			Callonetta leucophrys	KBARG043-07
			Tringa flavipes	KBARG051-07
			Thalassarche melanophris	KBARG059-07
			Anairetes flavirostris	KBARG067-07
			Caprimulgus longirostris	KBARG075-07
			Butorides striata	KBARG083-07
			Gallinula chloropus	KBARG091-07
			Sterna superciliaris	KBARG003-07
			Callonetta leucophrys	KBARG011-07
			Phalacrocorax brasilianus	KBARG019-07
			Cyanocompsa brissonii	KBARG027-07
			Passer domesticus	KBARG035-07
			Passer domesticus	KBARG042-07
			Accipiter erythronemius	KBARG050-07
			Tyrannus savana	KBARG058-07
			Griseotyrannus aurantioatrocristatus	KBARG066-07

			Amazonetta brasiliensis	KBARG074-07
			Phaetusa simplex	KBARG082-07
			Cyanocompsa brissonii	KBARG090-07
			Glaucidium brasilianum	KBARG002-07
			Buteo magnirostris	KBARG010-07
			Caprimulgus parvulus	KBARG018-07
			Pachyramphus validus	KBARG026-07
			Griseotyrannus aurantioatrocristatus	KBARG034-07
			Amazonetta brasiliensis	KBARG041-07
			Cathartes burrovianus	KBARG049-07
			Accipiter erythronemius	KBARG057-07
			Bubo magellanicus	KBARG065-07
			Troglodytes aedon	KBARG073-07
			Thlypopsis sordida	KBARG081-07
			Butorides striata	KBARG089-07
			Tyrannus melancholicus	KBARG001-07
			Amazonetta brasiliensis	KBARG009-07
			Tringa flavipes	KBARG017-07
			Tyrannus savana	KBARG025-07
			Larus dominicanus	KBARG033-07
			Saltator coerulescens	KBARG040-07
			Tigrisoma lineatum	KBARG048-07
			Platalea ajaja	KBARG056-07
			Pyrocephalus rubinus	KBARG064-07
			Platalea ajaja	KBARG072-07
			Calidris melanotos	KBARG080-07
			Phaetusa simplex	KBARG088-07
**Fishes**		187	Trachonurus sentipellis	FOAF256-07
			Lucigadus ori	FOAF267-07
			Scombrolabrax heterolepis	FOAF554-07
			Neoepinnula orientalis	FOAF541-07
			Coryphaenoides serrulatus	FOAF261-07
			Caelorinchus innotabilis	FOAF288-07
			Bathygadus spongiceps	FOAF274-07
			Tylerius spinosissimus	FOAF353-07
			Trachonurus yiwardaus	FOAF265-07
			Lucigadus ori	FOAF268-07
			Psenopsis obscura	FOAF472-07
			Rexea prometheoides	FOAF542-07
			Coryphaenoides serrulatus	FOAF262-07
			Caelorinchus innotabilis	FOAF289-07
			Caelorinchus acanthiger	FOAF290-07
			Tylerius spinosissimus	FOAF354-07
			Ventrifossa johnboborum	FOAF282-07
			Malacocephalus laevis	FOAF251-07
			Psenopsis obscura	FOAF473-07
			Rexea prometheoides	FOAF543-07
			Coryphaenoides serrulatus	FOAF263-07
			Caelorinchus maurofasciatus	FOAF296-07
			Caelorinchus acanthiger	FOAF291-07
			Pseudocaranx dentex	FOAF169-07
			Ventrifossa nigrodorsalis	FOAF283-07

			Malacocephalus laevis	FOAF252-07
			Psenopsis obscura	FOAF474-07
			Rexea prometheoides	FOAF544-07
			Coryphaenoides serrulatus	FOAF264-07
			Caelorinchus maurofasciatus	FOAF297-07
			Caelorinchus acanthiger	FOAF292-07
			Parequula sp.	FOAF166-07
			Callionymus cf. australis	FOAF442-07
			Malacocephalus laevis	FOAF253-07
			Cubiceps whiteleggi	FOAF475-07
			Rexea prometheoides	FOAF545-07
			Gadomus cf. colletti	FOAF277-07
			Caelorinchus maurofasciatus	FOAF298-07
			Caelorinchus acanthiger	FOAF293-07
			Parequula cf. melbournensis	FOAF170-07
			Callionymus cf. australis	FOAF443-07
			Malacocephalus laevis	FOAF254-07
			Cubiceps whiteleggi	FOAF476-07
			Rexea prometheoides	FOAF546-07
			Gadomus cf. colletti	FOAF278-07
			Caelorinchus maurofasciatus	FOAF299-07
			Caelorinchus acutirostris	FOAF306-07
			Parequula sp.	FOAF178-07
			Foetorepus apricus	FOAF437-07
			Nezumia propinqua	FOAF280-07
			Cubiceps whiteleggi	FOAF477-07
			Rexea solandri	FOAF547-07
			Gadomus cf. colletti	FOAF279-07
			Caelorinchus maurofasciatus	FOAF300-07
			Caelorinchus acutirostris	FOAF307-07
			Parequula sp.	FOAF179-07
			Foetorepus apricus	FOAF438-07
			Nezumia propinqua	FOAF281-07
			Gadomus pepperi	FOAF271-07
			Rexea solandri	FOAF548-07
			Gadomus pepperi	FOAF270-07
			Caelorinchus mirus	FOAF284-07
			Caelorinchus amydrozosterus	FOAF301-07
			Pseudorhombus jenynsii	FOAF173-07
			Foetorepus apricus	FOAF439-07
			Nezumia wularnia	FOAF269-07
			Lepidorhynchus denticulatus	FOAF257-07
			Rexea solandri	FOAF549-07
			Lepidocybium flavobrunneum	FOAF550-07
			Caelorinchus mirus	FOAF285-07
			Caelorinchus argentatus	FOAF312-07
			Anoplocapros amygdaloides	FOAF155-07
			Foetorepus apricus	FOAF440-07
			Nezumia wularnia	FOAF275-07
			Lepidorhynchus denticulatus	FOAF258-07
			Scombrolabrax heterolepis	FOAF551-07
			Neoepinnula orientalis	FOAF538-07

			Caelorinchus mirus	FOAF286-07
			Caelorinchus charius	FOAF294-07
			Bathygadus spongiceps	FOAF272-07
			Foetorepus apricus	FOAF441-07
			Nezumia wularnia	FOAF276-07
			Lepidorhynchus denticulatus	FOAF259-07
			Scombrolabrax heterolepis	FOAF552-07
			Neoepinnula orientalis	FOAF539-07
			Caelorinchus mirus	FOAF287-07
			Caelorinchus charius	FOAF295-07
			Bathygadus spongiceps	FOAF273-07
			Neatypus obliquus	FOAF172-07
			Trachonurus sentipellis	FOAF255-07
			Lepidorhynchus denticulatus	FOAF260-07
			Scombrolabrax heterolepis	FOAF553-07
			Neoepinnula orientalis	FOAF540-07
			Cetonurus globiceps	FOAF266-07
			Apristurus sp. D	FOAG046-07
			Apristurus sp. B	FOAG022-07
			Apristurus sp. G	FOAG057-07
			Centrophorus uyato	FOAG072-07
			Gymnura japonica	FOAF571-07
			Zeus faber	FOAF176-07
			Dasyatis brevicaudata	FOAF161-07
			Lepidoperca occidentalis	FOAF157-07
			Apristurus sp. D	FOAG047-07
			Apristurus sp. B	FOAG063-07
			Apristurus sp. G	FOAG058-07
			Centrophorus uyato	FOAG073-07
			Gymnura japonica	FOAF572-07
			Neoscorpaena cf. nielseni	FOAF214-07
			Dasyatis brevicaudata	FOAF162-07
			Zanclistius elevatus	FOAF153-07
			Apristurus sp. D	FOAG048-07
			Apristurus sp. B	FOAG064-07
			Apristurus sp. G	FOAG003-07
			Apristurus sp. E	FOAG012-07
			Gymnura japonica	FOAF573-07
			Sillaginodes punctatus	FOAF174-07
			Dasyatis brevicaudata	FOAF163-07
			Nemadactylus valenciennesi	FOAF156-07
			Apristurus sp. D	FOAG049-07
			Apristurus sp. C	FOAG034-07
			Apristurus sp. G	FOAG004-07
			Apristurus sp. E	FOAG013-07
			Gymnura japonica	FOAF574-07
			Centrophorus moluccensis	FOAF189-07
			Dasyatis brevicaudata	FOAF164-07
			Goniistius gibbosus	FOAF177-07
			Apristurus sp. D	FOAG050-07
			Apristurus sp. C	FOAG035-07
			Apristurus sp. G	FOAG005-07

			Apristurus sp. E	FOAG054-07
			Aetomylaeus nichofii	FOAF150-07
			Centrophorus moluccensis	FOAF190-07
			Sardinella lemuru	FOAF167-07
			Coris auricularis	FOAF175-07
			Apristurus sp. A	FOAG025-07
			Apristurus sp. C	FOAG036-07
			Apristurus sp. G	FOAG006-07
			Apristurus sp. E	FOAG055-07
			Centrophorus uyato	FOAG065-07
			Irolita waitii	FOAF180-07
			Sardinops sagax	FOAF168-07
			Himantolophus appelii	FOAF151-07
			Apristurus sp. A	FOAG026-07
			Apristurus sp. C	FOAG037-07
			Apristurus sp. G	FOAG007-07
			Apristurus sp. E	FOAG009-07
			Centrophorus uyato	FOAG066-07
			Trachinocephalus myops	FOAF185-07
			Cleidopus gloriamaris	FOAF165-07
			Gigantactis paxtoni	FOAF152-07
			Apristurus sp. A	FOAG028-07
			Apristurus sp. C	FOAG038-07
			Apristurus sp. G	FOAG008-07
			Apristurus sp. E	FOAG010-07
			Centrophorus uyato	FOAG067-07
			Bathymicrops cf. brevianalis	FOAF527-07
			Cyttopsis cypho	FOAF455-07
			Solegnathus lettiensis	FOAF154-07
			Apristurus sp. A	FOAG031-07
			Apristurus sp. C	FOAG039-07
			Apristurus sp. B	FOAG018-07
			Apristurus sp. E	FOAG011-07
			Centrophorus uyato	FOAG068-07
			Bathymicrops cf. brevianalis	FOAF524-07
			Centroberyx australis	FOAF465-07
			Solegnathus lettiensis	FOAF159-07
			Apristurus sp. A	FOAG032-07
			Apristurus sp. C	FOAG040-07
			Apristurus sp. B	FOAG019-07
			Apristurus sp. E	FOAG014-07
			Centrophorus uyato	FOAG069-07
			Bathymicrops cf. brevianalis	FOAF525-07
			Centroberyx australis	FOAF466-07
			Metavelifer multiradiatus	FOAF158-07
			Apristurus sp. A	FOAG060-07
			Apristurus sp. D	FOAG016-07
			Apristurus sp. B	FOAG020-07
			Apristurus sp. E	FOAG015-07
			Centrophorus uyato	FOAG070-07
			Bathymicrops cf. brevianalis	FOAF526-07
			Centroberyx australis	FOAF467-07

			Dasyatis brevicaudata	FOAF160-07
			Apristurus sp. D	FOAG017-07
			Apristurus sp. B	FOAG021-07
			Apristurus sp. G	FOAG056-07
			Centrophorus uyato	FOAG071-07
			Gymnura japonica	FOAF570-07
**Amphibians**		92	Rana catesbeiana	AMPAS178-05
			Plethodon cinereus	AMPAS166-05
			Rana clamitans	AMPAS154-05
			Rana catesbeiana	AMPAS142-05
			Rana septentrionalis	AMPAS130-05
			Rana pipiens	AMPAS118-05
			Hyla versicolor	AMPAS107-05
			Bufo americanus	AMPAS096-05
			Rana catesbeiana	AMPAS179-05
			Plethodon cinereus	AMPAS167-05
			Rana clamitans	AMPAS188-05
			Rana catesbeiana	AMPAS143-05
			Rana catesbeiana	AMPAS131-05
			Rana clamitans	AMPAS119-05
			Hyla versicolor	AMPAS108-05
			Bufo americanus	AMPAS097-05
			Bufo fowleri	AMPAS180-05
			Plethodon cinereus	AMPAS168-05
			Ambystoma laterale-jeffersonianum complex	AMPAS156-05
			Rana pipiens	AMPAS144-05
			Rana septentrionalis	AMPAS132-05
			Rana pipiens	AMPAS120-05
			Hyla versicolor	AMPAS109-05
			Bufo americanus	AMPAS098-05
			Bufo fowleri	AMPAS181-05
			Hemidactylium scutatum	AMPAS169-05
			Ambystoma laterale-jeffersonianum complex	AMPAS157-05
			Rana clamitans	AMPAS145-05
			Rana septentrionalis	AMPAS133-05
			Rana clamitans	AMPAS121-05
			Hyla versicolor	AMPAS110-05
			Bufo americanus	AMPAS099-05
			Bufo fowleri	AMPAS182-05
			Plethodon cinereus	AMPAS170-05
			Ambystoma laterale-jeffersonianum complex	AMPAS158-05
			Rana catesbeiana	AMPAS146-05
			Rana septentrionalis	AMPAS134-05
			Rana sylvatica	AMPAS122-05
			Hyla versicolor	AMPAS111-05
			Bufo americanus	AMPAS100-05
			Pseudacris triseriata	AMPAS183-05
			Notophthalmus viridescens	AMPAS171-05
			Ambystoma laterale-jeffersonianum complex	AMPAS159-05
			Rana catesbeiana	AMPAS147-05
			Rana sylvatica	AMPAS135-05
			Rana sylvatica	AMPAS123-05

			Rana clamitans	AMPAS112-05
			Bufo americanus	AMPAS101-05
			Pseudacris triseriata	AMPAS184-05
			Rana pipiens	AMPAS172-05
			Plethodon cinereus	AMPAS160-05
			Rana clamitans	AMPAS148-05
			Rana septentrionalis	AMPAS136-05
			Rana pipiens	AMPAS124-05
			Rana clamitans	AMPAS113-05
			Bufo americanus	AMPAS102-05
			Rana sylvatica	AMPAS185-05
			Bufo americanus	AMPAS173-05
			Plethodon cinereus	AMPAS161-05
			Rana pipiens	AMPAS149-05
			Rana clamitans	AMPAS137-05
			Rana sylvatica	AMPAS125-05
			Rana clamitans	AMPAS114-05
			Bufo americanus	AMPAS103-05
			Rana sylvatica	AMPAS186-05
			Rana	AMPAS174-05
			Plethodon cinereus	AMPAS162-05
			Rana pipiens	AMPAS150-05
			Rana sylvatica	AMPAS138-05
			Rana sylvatica	AMPAS126-05
			Rana clamitans	AMPAS115-05
			Pseudacris crucifer	AMPAS104-05
			Pseudacris crucifer	AMPAS187-05
			Rana sylvatica	AMPAS175-05
			Hemidactylium scutatum	AMPAS163-05
			Rana pipiens	AMPAS151-05
			Rana sylvatica	AMPAS139-05
			Rana clamitans	AMPAS127-05
			Pseudacris crucifer	AMPAS105-05
			Hyla versicolor	AMPAS095-05
			Rana	AMPAS176-05
			Plethodon cinereus	AMPAS164-05
			Rana sylvatica	AMPAS152-05
			Rana septentrionalis	AMPAS140-05
			Rana sylvatica	AMPAS128-05
			Rana pipiens	AMPAS117-05
			Pseudacris crucifer	AMPAS106-05
			Rana catesbeiana	AMPAS177-05
			Plethodon cinereus	AMPAS165-05
			Rana clamitans	AMPAS153-05
			Rana pipiens	AMPAS141-05
			Rana	AMPAS129-05
**Mollusks**		94	Columbellidae	MBMIB271-06
			Galeommatidae	MBMIB259-06
			Glossodoris sibogae	MBMIB247-06
			Conus distans	MBMIB235-06
			Drupella cornus	MBMIB223-06
			Terebra affinis	MBMIB211-06

			Rhinoclavis fasciata	MBMIB200-06
			Botula sp.	MBMIB189-06
			Columbellidae	MBMIB272-06
			Berthelinia citrina	MBMIB260-06
			Aspella anceps	MBMIB248-06
			Cypraea helvola	MBMIB236-06
			Drupella cornus	MBMIB224-06
			Nassarius graniferus	MBMIB212-06
			Cerithium salebrosum	MBMIB201-06
			Melampus sp.	MBMIB190-06
			Conus leopardus	MBMIB273-06
			Berthelinia citrina	MBMIB261-06
			Maculotriton serriale	MBMIB249-06
			Pascahinnites coruscans	MBMIB237-06
			Drupella cornus	MBMIB225-06
			Terebra subulata	MBMIB213-06
			Rhinoclavis fasciata	MBMIB202-06
			Melampus sp.	MBMIB191-06
			Conus leopardus	MBMIB274-06
			Elysia sp.	MBMIB262-06
			Buccinidae	MBMIB250-06
			Pascahinnites coruscans	MBMIB238-06
			Conus ebraeus	MBMIB226-06
			Gafrarium pectinatum	MBMIB214-06
			Terebra affinis	MBMIB203-06
			Fragum fragum	MBMIB192-06
			Strombus lentiginosus	MBMIB275-06
			Otopleura sp.	MBMIB263-06
			Galeommatidae	MBMIB251-06
			Trochidae	MBMIB239-06
			Arca avellana	MBMIB227-06
			Lithophaga nigra	MBMIB215-06
			Strombus gibberulus	MBMIB204-06
			Terebra cingulifera	MBMIB193-06
			Anomiidae	MBMIB276-06
			Pupa sp.	MBMIB264-06
			Muricidae	MBMIB252-06
			Muricidae	MBMIB240-06
			Arca avellana	MBMIB228-06
			Chama asperella	MBMIB216-06
			Pupa solidula	MBMIB205-06
			Imbricaria olivaeformis	MBMIB194-06
			Isognomon sp.	MBMIB277-06
			Lamellaria sp.	MBMIB265-06
			Muricidae	MBMIB253-06
			Muricidae	MBMIB241-06
			Conus lividus	MBMIB229-06
			Limaria fragilis	MBMIB217-06
			Rhinoclavis fasciata	MBMIB206-06
			Terebra affinis	MBMIB195-06
			Lithophaga sp.	MBMIB278-06
			Notaspidea	MBMIB266-06

			Conus miliaris	MBMIB254-06
			Planaxis sp.	MBMIB242-06
			Isognomon sp.	MBMIB230-06
			Limaria fragilis	MBMIB218-06
			Otopleura mitralis	MBMIB207-06
			Neocancilla papilio	MBMIB196-06
			Lithophaga sp.	MBMIB279-06
			Opisthobranchia	MBMIB267-06
			Lithophaga sp.	MBMIB255-06
			Cymatium sp.	MBMIB243-06
			Cypraea helvola	MBMIB231-06
			Limaria fragilis	MBMIB219-06
			Terebra affinis	MBMIB208-06
			Strombus gibberulus	MBMIB197-06
			Polyplacophora	MBMIB280-06
			Opisthobranchia	MBMIB268-06
			Lithophaga sp.	MBMIB256-06
			Planaxis sp.	MBMIB244-06
			Parahyotissa numisma	MBMIB232-06
			Vanikoro sp.	MBMIB220-06
			Cerithium salebrosum	MBMIB209-06
			Nassarius graniferus	MBMIB198-06
			Nassarius graniferus	MBMIB281-06
			Galeommatidae	MBMIB269-06
			Atlantidae	MBMIB257-06
			Sabia conica	MBMIB245-06
			Drupella ochrostoma	MBMIB233-06
			Drupa grossularia	MBMIB221-06
			Nassarius graniferus	MBMIB210-06
			Cerithium salebrosum	MBMIB199-06
			Cerithium salebrosum	MBMIB282-06
			Columbellidae	MBMIB270-06
			Octopus bocki	MBMIB258-06
			Conus imperialis	MBMIB246-06
			Drupella ochrostoma	MBMIB234-06
			Arca ventricosa	MBMIB222-06
**Crustaceans**		93	Stenopus hispidus	MBMIA637-06
			Xanthidae	MBMIA625-06
			Uca sp.	MBMIA613-06
			Harpiliopsis sp.	MBMIA601-06
			Alpheus sp.	MBMIA589-06
			Menaethius monoceros	MBMIA578-06
			Coenobita perlatus	MBMIA567-06
			Grapsus tenuicrustatus	MBMIA556-06
			Thalamita coeruleipes	MBMIA638-06
			Alpheus sp.	MBMIA626-06
			Uca tetragonon	MBMIA614-06
			Metopograpsus thukuhar	MBMIA602-06
			Alpheus sp.	MBMIA590-06
			Thalamita sp.	MBMIA579-06
			Coenobita rugosus	MBMIA568-06
			Grapsus tenuicrustatus	MBMIA557-06

			Etisus dentatus	MBMIA639-06
			Alpheus sp.	MBMIA627-06
			Uca chlorophthalmus	MBMIA615-06
			Metopograpsus thukuhar	MBMIA603-06
			Xanthidae	MBMIA591-06
			Trapezia serenei	MBMIA580-06
			Calcinus seurati	MBMIA569-06
			Geograpsus crinipes	MBMIA558-06
			Pilodius sp.	MBMIA640-06
			Alpheus sp.	MBMIA628-06
			Uca tetragonon	MBMIA616-06
			Metopograpsus thukuhar	MBMIA604-06
			Cyclodius ungulatus	MBMIA592-06
			Isopoda	MBMIA581-06
			Calcinus seurati	MBMIA570-06
			Geograpsus crinipes	MBMIA559-06
			Sesarminae	MBMIA641-06
			Alpheus sp.	MBMIA629-06
			Uca tetragonon	MBMIA617-06
			Galatheidae	MBMIA605-06
			Liomera bella	MBMIA593-06
			Trapezia sp.	MBMIA582-06
			Calcinus seurati	MBMIA571-06
			Charybdis obtusifrons	MBMIA560-06
			Saron sp.	MBMIA642-06
			Xanthidae	MBMIA630-06
			Uca chlorophthalmus	MBMIA618-06
			Perinea tumida	MBMIA606-06
			Pseudoliomera remota	MBMIA594-06
			Trapezia rufopunctata	MBMIA583-06
			Pseudozius caystrus	MBMIA572-06
			Percnon planissimus	MBMIA561-06
			Nikoides sp.	MBMIA643-06
			Xanthidae	MBMIA631-06
			Uca sp.	MBMIA619-06
			Xanthidae	MBMIA607-06
			Cyclodius ungulatus	MBMIA595-06
			Trapezia rufopunctata	MBMIA584-06
			Xanthidae	MBMIA573-06
			Percnon planissimus	MBMIA562-06
			Nikoides sp.	MBMIA644-06
			Paractaea rufopunctata	MBMIA632-06
			Raoulserenea sp.	MBMIA620-06
			Uca tetragonon	MBMIA608-06
			Neoliomera sp.	MBMIA596-06
			Calcinus elegans	MBMIA574-06
			Percnon planissimus	MBMIA563-06
			Nikoides sp.	MBMIA645-06
			Pilodius pugil	MBMIA633-06
			Raoulserenea sp.	MBMIA621-06
			Uca chlorophthalmus	MBMIA609-06
			Trapezia areolata	MBMIA597-06

			Alpheus sp.	MBMIA585-06
			Pachygrapsus sp.	MBMIA575-06
			Percnon planissimus	MBMIA564-06
			Lissocarcinus orbicularis	MBMIA646-06
			Pilumnus sp.	MBMIA634-06
			Axiidae	MBMIA622-06
			Uca chlorophthalmus	MBMIA610-06
			Dynomene sp.	MBMIA598-06
			Alpheus sp.	MBMIA586-06
			Pachygrapsus sp.	MBMIA576-06
			Leptodius sp.	MBMIA565-06
			Nikoides sp.	MBMIA647-06
			Pilumnus sp.	MBMIA635-06
			Majidae	MBMIA623-06
			Uca chlorophthalmus	MBMIA611-06
			Trapezia serenei	MBMIA599-06
			Alpheus sp.	MBMIA587-06
			Pachygrapsus sp.	MBMIA577-06
			Coenobita perlatus	MBMIA566-06
			Trapezia punctimanus	MBMIA648-06
			Stenopus hispidus	MBMIA636-06
			Xanthidae	MBMIA624-06
			Uca sp.	MBMIA612-06
			Trapezia sp.	MBMIA600-06
			Alpheus sp.	MBMIA588-06
**Insects**	EPT	270	Rhyacophila carolina	SMCAD659-07
			Lepidostoma pictile	SMCAD660-07
			Lepidostoma pictile	SMCAD661-07
			Lepidostoma pictile	SMCAD662-07
			Lepidostoma pictile	SMCAD663-07
			Lepidostoma pictile	SMCAD664-07
			Lepidostoma sp.	SMCAD665-07
			Lepidostoma sp.	SMCAD666-07
			Lepidostoma sp.	SMCAD667-07
			Hydropsyche sp.	SMCAD668-07
			Cheumatopsyche sp.	SMCAD669-07
			Brachycentrus sp.	SMCAD670-07
			Nyctiophylax nephophilus	SMCAD671-07
			Nyctiophylax nephophilus	SMCAD672-07
			Nyctiophylax nephophilus	SMCAD673-07
			Ceratopsyche sparna	SMCAD674-07
			Ceratopsyche sparna	SMCAD675-07
			Lepidostoma pictile	SMCAD676-07
			Lepidostoma pictile	SMCAD677-07
			Lepidostoma pictile	SMCAD678-07
			Dolophilodes distincta	SMCAD679-07
			Dolophilodes distincta	SMCAD680-07
			Goera calcarata	SMCAD681-07
			Goera calcarata	SMCAD682-07
			Goera calcarata	SMCAD683-07
			Nyctiophylax sp.	SMCAD684-07
			Nyctiophylax sp.	SMCAD685-07

			Nyctiophylax sp.	SMCAD686-07
			Dolophilodes distincta	SMCAD687-07
			Dolophilodes distincta	SMCAD688-07
			Stactobiella martynovi	SMCAD689-07
			Fumonta major	SMCAD690-07
			Fumonta major	SMCAD691-07
			Fumonta major	SMCAD692-07
			Goera calcarata	SMCAD693-07
			Ceratopsyche sp.	SMCAD694-07
			Ceratopsyche sp.	SMCAD695-07
			Ceratopsyche sp.	SMCAD696-07
			Agapetus tomus	SMCAD697-07
			Lype diversa	SMCAD698-07
			Cheumatopsyche sp.	SMCAD699-07
			Agapetus sp.	SMCAD700-07
			Psychomyia flavida	SMCAD701-07
			Psychomyia flavida	SMCAD702-07
			Psychomyia flavida	SMCAD703-07
			Psychomyia flavida	SMCAD704-07
			Psychomyia flavida	SMCAD705-07
			Lepidostoma sp.	SMCAD706-07
			Hydropsychidae	SMCAD707-07
			Ceratopsyche sparna	SMCAD708-07
			Ceratopsyche sparna	SMCAD709-07
			Ceratopsyche sparna	SMCAD710-07
			Ceratopsyche sparna	SMCAD711-07
			Micrasema wataga	SMCAD712-07
			Micrasema wataga	SMCAD713-07
			Lepidostoma sp.	SMCAD714-07
			Hydroptilidae	SMCAD715-07
			Hydroptilidae	SMCAD716-07
			Hydroptilidae	SMCAD717-07
			Hydroptilidae	SMCAD718-07
			Lepidostoma sp.	SMCAD719-07
			Cheumatopsyche sp.	SMCAD720-07
			Agapetus walkeri	SMCAD721-07
			Micrasema sp.	SMCAD722-07
			Micrasema sp.	SMCAD723-07
			Micrasema sp.	SMCAD724-07
			Stactobiella sp.	SMCAD725-07
			Stactobiella sp.	SMCAD726-07
			Stactobiella sp.	SMCAD727-07
			Stactobiella sp.	SMCAD728-07
			Stactobiella sp.	SMCAD729-07
			Rhyacophila fuscula	SMCAD730-07
			Rhyacophila fuscula	SMCAD731-07
			Ceratopsyche sparna	SMCAD732-07
			Ceratopsyche sparna	SMCAD733-07
			Ceratopsyche bronta	SMCAD734-07
			Ceratopsyche bronta	SMCAD735-07
			Ceratopsyche morosa	SMCAD736-07
			Polycentropus confusus	SMCAD737-07

			Goera calcarata	SMCAD738-07
			Mystacides sepulchralis	SMCAD739-07
			Dolophilodes distincta	SMCAD740-07
			Dolophilodes distincta	SMCAD741-07
			Rhyacophila nigrita	SMCAD742-07
			Neophylax mitchelli	SMCAD743-07
			Neophylax mitchelli	SMCAD744-07
			Psilotreta amera	SMCAD745-07
			Psilotreta amera	SMCAD746-07
			Psilotreta amera	SMCAD747-07
			Pycnopsyche gentilis	SMCAD748-07
			Pycnopsyche gentilis	SMCAD749-07
			Agapetus sp.	SMCAD750-07
			Parapsyche cardis	SMCAD751-07
			Parapsyche cardis	SMCAD752-07
			Acentrella sp.	SMMAY096-07
			Acentrella sp.	SMMAY097-07
			Acentrella sp.	SMMAY098-07
			Acentrella sp.	SMMAY099-07
			Baetisca carolina	SMMAY100-07
			Baetisca carolina	SMMAY101-07
			Baetisca carolina	SMMAY102-07
			Maccaffertium ithaca	SMMAY104-07
			Maccaffertium ithaca	SMMAY105-07
			Maccaffertium ithaca	SMMAY106-07
			Epeorus sp.	SMMAY107-07
			Epeorus sp.	SMMAY108-07
			Rhithrogena sp.	SMMAY109-07
			Baetisca intercalaris	SMMAY110-07
			Procloeon sp.	SMMAY112-07
			Procloeon sp.	SMMAY113-07
			Stenacron interpunctatum	SMMAY114-07
			Stenacron interpunctatum	SMMAY115-07
			Habrophlebia vibrans	SMMAY116-07
			Pseudocloeon sp.	SMMAY117-07
			Epeorus sp.	SMMAY118-07
			Epeorus sp.	SMMAY120-07
			Baetis pluto	SMMAY121-07
			Baetis pluto	SMMAY122-07
			Drunella tuberculata	SMMAY123-07
			Dannella provolshia	SMMAY124-07
			Dannella provolshia	SMMAY125-07
			Acentrella turbida	SMMAY126-07
			Acentrella barbarae	SMMAY128-07
			Acentrella barbarae	SMMAY129-07
			Ephemerella catawba	SMMAY130-07
			Ephemerella catawba	SMMAY131-07
			Paraleptophlebia guttata	SMMAY132-07
			Ephemerella sp.	SMMAY133-07
			Ephemerella sp.	SMMAY134-07
			Ephemerella sp.	SMMAY136-07
			Ephemerella sp.	SMMAY137-07

			Drunella walkeri	SMMAY138-07
			Drunella walkeri	SMMAY139-07
			Ameletus sp.	SMMAY140-07
			Baetisca carolina	SMMAY141-07
			Baetisca carolina	SMMAY142-07
			Epeorus sp.	SMMAY144-07
			Epeorus sp.	SMMAY145-07
			Paraleptophlebia assimilis	SMMAY146-07
			Paraleptophlebia assimilis	SMMAY147-07
			Ephemerella excrucians	SMMAY148-07
			Neoephemera purpurea	SMMAY149-07
			Neoephemera purpurea	SMMAY150-07
			Baetis flavistriga	SMMAY152-07
			Baetis flavistriga	SMMAY153-07
			Acentrella turbida	SMMAY154-07
			Acentrella turbida	SMMAY155-07
			Acentrella turbida	SMMAY156-07
			Leucrocuta sp2	SMMAY157-07
			Leucrocuta sp1	SMMAY158-07
			Ephemerella dorothea	SMMAY160-07
			Centroptilum sp.	SMMAY161-07
			Ephemerella sp.	SMMAY162-07
			Dannella provolshia	SMMAY163-07
			Eurylophella sp.	SMMAY164-07
			Eurylophella sp.	SMMAY165-07
			Eurylophella sp.	SMMAY166-07
			Plauditus sp.	SMMAY168-07
			Plauditus sp.	SMMAY169-07
			Maccaffertium sp.	SMMAY170-07
			Maccaffertium sp.	SMMAY171-07
			Isonychia tusculanensis	SMMAY172-07
			Isonychia tusculanensis	SMMAY173-07
			Baetis pluto	SMMAY174-07
			Baetis pluto	SMMAY176-07
			Baetis pluto	SMMAY177-07
			Baetis intercalaris	SMMAY178-07
			Leucrocuta aphrodite	SMMAY179-07
			Eurylophella sp.	SMMAY180-07
			Leucrocuta aphrodite	SMMAY181-07
			Leucrocuta aphrodite	SMMAY182-07
			Baetis pluto	SMMAY184-07
			Baetis pluto	SMMAY185-07
			Baetis pluto	SMMAY186-07
			Baetis pluto	SMMAY187-07
			Baetis pluto	SMMAY188-07
			Acroneuria abnormis	SMSTO001-07
			Acroneuria abnormis	SMSTO002-07
			Isoperla holochlora	SMSTO003-07
			Isoperla dicala	SMSTO004-07
			Neoperla sp.	SMSTO005-07
			Neoperla sp.	SMSTO006-07
			Perlesta sp. 1	SMSTO007-07

			Perlesta sp. 1	SMSTO008-07
			Paragnetina media	SMSTO009-07
			Perlesta sp. 2	SMSTO010-07
			Perlesta sp. 2	SMSTO011-07
			Isoperla sp.	SMSTO012-07
			Isoperla sp.	SMSTO013-07
			Acroneuria abnormis	SMSTO014-07
			Acroneuria abnormis	SMSTO015-07
			Pteronarcys sp.	SMSTO016-07
			Isoperla holochlora	SMSTO017-07
			Isoperla holochlora	SMSTO018-07
			Alloperla sp.	SMSTO019-07
			Alloperla sp.	SMSTO020-07
			Paragnetina media	SMSTO021-07
			Paragnetina media	SMSTO022-07
			Alloperla atlantica	SMSTO023-07
			Alloperla atlantica	SMSTO024-07
			Acroneuria filicis	SMSTO025-07
			Acroneuria filicis	SMSTO026-07
			Sweltsa mediana	SMSTO027-07
			Amphinemura nigritta	SMSTO028-07
			Amphinemura nigritta	SMSTO029-07
			Perlesta sp. 1	SMSTO030-07
			Perlesta sp. 1	SMSTO031-07
			Tallaperla sp.	SMSTO032-07
			Tallaperla sp.	SMSTO033-07
			Remenus bilobatus	SMSTO034-07
			Remenus bilobatus	SMSTO035-07
			Amphinemura wui	SMSTO036-07
			Amphinemura wui	SMSTO037-07
			Acroneuria abnormis	SMSTO038-07
			Pteronarcys sp.	SMSTO039-07
			Pteronarcys sp.	SMSTO040-07
			Beloneuria sp.	SMSTO041-07
			Isoperla holochlora	SMSTO042-07
			Isoperla sp.	SMSTO043-07
			Isoperla sp.	SMSTO044-07
			Alloperla atlantica	SMSTO045-07
			Alloperla atlantica	SMSTO046-07
			Sweltsa mediana	SMSTO047-07
			Haploperla brevis	SMSTO048-07
			Haploperla brevis	SMSTO049-07
			Amphinemura wui	SMSTO050-07
			Isoperla namata	SMSTO051-07
			Isoperla holochlora	SMSTO052-07
			Pteronarcys sp. 2	SMSTO053-07
			Pteronarcys sp. 2	SMSTO054-07
			Isoperla holochlora	SMSTO055-07
			Isoperla holochlora	SMSTO056-07
			Tallaperla sp.	SMSTO057-07
			Tallaperla sp.	SMSTO058-07
			Isoperla namata	SMSTO059-07

			Isoperla namata	SMSTO060-07
			Haploperla sp.	SMSTO061-07
			Haploperla sp.	SMSTO062-07
			Acroneuria abnormis	SMSTO063-07
			Acroneuria abnormis	SMSTO064-07
			Eccoptura xanthenes	SMSTO065-07
			Eccoptura xanthenes	SMSTO066-07
			Tallaperla sp. 2	SMSTO067-07
			Alloperla atlantica	SMSTO068-07
			Sweltsa mediana	SMSTO069-07
			Sweltsa mediana	SMSTO070-07
			Amphinemura sp.	SMSTO071-07
			Amphinemura sp.	SMSTO072-07
			Haploperla brevis	SMSTO073-07
			Remenus bilobatus	SMSTO074-07
			Acroneuria abnormis	SMSTO075-07
			Acroneuria abnormis	SMSTO076-07
			Isoperla holochlora	SMSTO077-07
			Isoperla holochlora	SMSTO078-07
			Neoperla sp.	SMSTO079-07
			Neoperla sp.	SMSTO080-07
			Neoperla sp.	SMSTO081-07
			Neoperla sp.	SMSTO082-07
			Amphinemura wui	SMSTO083-07
			Remenus bilobatus	SMSTO084-07
			Remenus bilobatus	SMSTO085-07
			Isoperla holochlora	SMSTO086-07
			Isoperla holochlora	SMSTO087-07
			Pteronarcys sp.	SMSTO088-07
			Pteronarcys sp.	SMSTO089-07
			Isoperla dicala	SMSTO090-07
			Acroneuria abnormis	SMSTO091-07
			Acroneuria abnormis	SMSTO092-07
			Acroneuria filicis	SMSTO093-07
			Acroneuria filicis	SMSTO094-07
	Wasps	93	Taeniogonalos gundlachii	ASTR052-06
			Taeniogonalos gundlachii	ASTR040-06
			Taeniogonalos gundlachii	ASTR028-06
			Taeniogonalos gundlachii	ASTR016-06
			Taeniogonalos gundlachii	ASTR004-06
			ichjanzen01 DHJPAR0010542	AICC967-06
			ichjanzen01 DHJPAR0010531	AICC956-06
			ichjanzen01 DHJPAR0010520	AICC945-06
			Taeniogonalos gundlachii	ASTR053-06
			Taeniogonalos gundlachii	ASTR041-06
			Taeniogonalos gundlachii	ASTR029-06
			Taeniogonalos gundlachii	ASTR017-06
			Taeniogonalos gundlachii	ASTR005-06
			ichjanzen01 DHJPAR0010543	AICC968-06
			ichjanzen01 DHJPAR0010532	AICC957-06
			ichjanzen01 DHJPAR0010521	AICC946-06
			Taeniogonalos gundlachii	ASTR054-06

			Taeniogonalos gundlachii	ASTR042-06
			Taeniogonalos gundlachii	ASTR030-06
			Taeniogonalos gundlachii	ASTR018-06
			Taeniogonalos gundlachii	ASTR006-06
			ichjanzen01 DHJPAR0010544	AICC969-06
			ichjanzen01 DHJPAR0010533	AICC958-06
			ichjanzen01 DHJPAR0010522	AICC947-06
			Taeniogonalos gundlachii	ASTR055-06
			Taeniogonalos gundlachii	ASTR043-06
			Taeniogonalos gundlachii	ASTR031-06
			Taeniogonalos gundlachii	ASTR019-06
			Taeniogonalos gundlachii	ASTR007-06
			ichjanzen01 DHJPAR0010545	AICC970-06
			ichjanzen01 DHJPAR0010534	AICC959-06
			ichjanzen01 DHJPAR0010523	AICC948-06
			Taeniogonalos gundlachii	ASTR056-06
			Taeniogonalos gundlachii	ASTR044-06
			Taeniogonalos gundlachii	ASTR032-06
			Taeniogonalos gundlachii	ASTR020-06
			Taeniogonalos gundlachii	ASTR008-06
			ichjanzen01 DHJPAR0010546	AICC971-06
			ichjanzen01 DHJPAR0010535	AICC960-06
			ichjanzen01 DHJPAR0010524	AICC949-06
			Taeniogonalos gundlachii	ASTR045-06
			Taeniogonalos gundlachii	ASTR033-06
			Taeniogonalos gundlachii	ASTR021-06
			Taeniogonalos gundlachii	ASTR009-06
			ichjanzen01 DHJPAR0010547	AICC972-06
			ichjanzen01 DHJPAR0010536	AICC961-06
			ichjanzen01 DHJPAR0010525	AICC950-06
			Taeniogonalos gundlachii	ASTR058-06
			Taeniogonalos gundlachii	ASTR046-06
			Taeniogonalos gundlachii	ASTR034-06
			Taeniogonalos gundlachii	ASTR022-06
			Taeniogonalos gundlachii	ASTR010-06
			ichjanzen01 DHJPAR0010548	AICC973-06
			ichjanzen01 DHJPAR0010537	AICC962-06
			ichjanzen01 DHJPAR0010526	AICC951-06
			Taeniogonalos gundlachii	ASTR059-06
			Taeniogonalos gundlachii	ASTR047-06
			Taeniogonalos gundlachii	ASTR035-06
			Taeniogonalos gundlachii	ASTR023-06
			Taeniogonalos gundlachii	ASTR011-06
			ichjanzen01 DHJPAR0010549	AICC974-06
			ichjanzen01 DHJPAR0010538	AICC963-06
			ichjanzen01 DHJPAR0010527	AICC952-06
			Taeniogonalos gundlachii	ASTR060-06
			Taeniogonalos gundlachii	ASTR048-06
			Taeniogonalos gundlachii	ASTR036-06
			Taeniogonalos gundlachii	ASTR024-06
			Taeniogonalos gundlachii	ASTR012-06
			ichjanzen01 DHJPAR0010550	AICC975-06

			ichjanzen01 DHJPAR0010539	AICC964-06
			ichjanzen01 DHJPAR0010528	AICC953-06
			Taeniogonalos gundlachii	ASTR061-06
			Taeniogonalos gundlachii	ASTR049-06
			Taeniogonalos gundlachii	ASTR037-06
			Taeniogonalos gundlachii	ASTR025-06
			Taeniogonalos gundlachii	ASTR013-06
			Taeniogonalos gundlachii	ASTR001-06
			ichjanzen01 DHJPAR0010540	AICC965-06
			ichjanzen01 DHJPAR0010529	AICC954-06
			Taeniogonalos gundlachii	ASTR062-06
			Taeniogonalos gundlachii	ASTR050-06
			Taeniogonalos gundlachii	ASTR038-06
			Taeniogonalos gundlachii	ASTR026-06
			Taeniogonalos gundlachii	ASTR014-06
			Taeniogonalos gundlachii	ASTR002-06
			ichjanzen01 DHJPAR0010541	AICC966-06
			ichjanzen01 DHJPAR0010530	AICC955-06
			Taeniogonalos gundlachii	ASTR063-06
			Taeniogonalos gundlachii	ASTR051-06
			Taeniogonalos gundlachii	ASTR039-06
			Taeniogonalos gundlachii	ASTR027-06
			Taeniogonalos gundlachii	ASTR015-06
			Taeniogonalos gundlachii	ASTR003-06
	Ants	94	Monomorium	ASAMX003-06
			Monomorium	ASAMX002-06
			Mystrium	ASAMX539-06
			Monomorium	ASAMX295-06
			Monomorium	ASAMX294-06
			Monomorium	ASAMX300-06
			Monomorium	ASAMX293-06
			Monomorium	ASAMX292-06
			Pachycondyla sp. jcr-09	ASAMX056-06
			Pachycondyla sp. jcr-09	ASAMX063-06
			Pachycondyla sikorae	ASAMX022-06
			Solenopsis	ASAMX225-06
			Monomorium	ASAMX001-06
			Monomorium	ASAMX006-06
			Monomorium	ASAMX005-06
			Monomorium	ASAMX004-06
			Pachycondyla ambigua	ASAMX205-06
			Pachycondyla ambigua	ASAMX173-06
			Cerapachys	ASAMX661-06
			Pachycondyla ambigua	ASAMX193-06
			Pachycondyla ambigua	ASAMX194-06
			Cerapachys	ASAMX663-06
			Cerapachys	ASAMX662-06
			Pachycondyla ambigua	ASAMX136-06
			Oligomyrmex	ASAMX247-06
			Oligomyrmex	ASAMX246-06
			Oligomyrmex	ASAMX245-06
			Oligomyrmex	ASAMX170-06

			Oligomyrmex	ASAMX017-06
			Oligomyrmex	ASAMX260-06
			Pachycondyla cambouei	ASAMX353-06
			Pachycondyla ambigua	ASAMX106-06
			Oligomyrmex	ASAMX104-06
			Oligomyrmex	ASAMX103-06
			Oligomyrmex	ASAMX102-06
			Oligomyrmex	ASAMX101-06
			Oligomyrmex	ASAMX068-06
			Oligomyrmex	ASAMX257-06
			Oligomyrmex	ASAMX190-06
			Oligomyrmex	ASAMX189-06
			Oligomyrmex	ASAMX172-06
			Oligomyrmex	ASAMX171-06
			Oligomyrmex	ASAMX192-06
			Oligomyrmex	ASAMX191-06
			Oligomyrmex	ASAMX135-06
			Oligomyrmex	ASAMX134-06
			Oligomyrmex	ASAMX133-06
			Oligomyrmex	ASAMX105-06
			Oligomyrmex	ASAMX150-06
			Oligomyrmex	ASAMX132-06
			Oligomyrmex	ASAMX131-06
			Oligomyrmex	ASAMX130-06
			Oligomyrmex	ASAMX129-06
			Oligomyrmex	ASAMX027-06
			Oligomyrmex	ASAMX026-06
			Oligomyrmex	ASAMX025-06
			Plagiolepis	ASAMX312-06
			Plagiolepis	ASAMX311-06
			Plagiolepis	ASAMX049-06
			Plagiolepis	ASAMX048-06
			Simopone	ASAMX340-06
			Simopone	ASAMX339-06
			Oligomyrmex	ASAMX152-06
			Oligomyrmex	ASAMX151-06
			Crematogaster	ASAMX303-06
			Crematogaster	ASAMX334-06
			Crematogaster	ASAMX333-06
			Crematogaster	ASAMX328-06
			Crematogaster	ASAMX314-06
			Crematogaster	ASAMX313-06
			Crematogaster	ASAMX332-06
			Crematogaster	ASAMX331-06
			Crematogaster	ASAMX316-06
			Crematogaster	ASAMX315-06
			Crematogaster	ASAMX348-06
			Crematogaster	ASAMX347-06
			Crematogaster	ASAMX336-06
			Crematogaster	ASAMX335-06
			Crematogaster	ASAMX317-06
			Crematogaster	ASAMX304-06

			Crematogaster	ASAMX067-06
			Crematogaster	ASAMX065-06
			Crematogaster	ASAMX064-06
			Crematogaster	ASAMX070-06
			Crematogaster	ASAMX289-06
			Crematogaster	ASAMX290-06
			Crematogaster	ASAMX288-06
			Crematogaster	ASAMX352-06
			Tetramorium	ASAMX237-06
			Tetramorium	ASAMX265-06
			Tetramorium	ASAMX264-06
			Tetramorium	ASAMX263-06
			Crematogaster	ASAMX024-06
			Crematogaster	ASAMX023-06
	Flies	94	Eucelatoria aurea	ASTAR553-07
			Eucelatoria aurea	ASTAR541-07
			Eucelatoria armigeraDHJ05	ASTAR529-07
			Calolydella Wood04	ASTAR517-07
			Calolydella Wood04	ASTAR505-07
			Calolydella Wood01	ASTAR493-07
			Calolydella Wood01	ASTAR482-07
			Nemorilla Wood01	ASTAR471-07
			Eucelatoria aurea	ASTAR554-07
			Eucelatoria aurea	ASTAR542-07
			Eucelatoria Wood01	ASTAR530-07
			Calolydella Wood04	ASTAR518-07
			Calolydella Wood04	ASTAR506-07
			Calolydella Wood01	ASTAR494-07
			Calolydella Wood01	ASTAR483-07
			Nemorilla Wood01	ASTAR472-07
			Eucelatoria aurea	ASTAR555-07
			Eucelatoria aurea	ASTAR543-07
			Eucelatoria Wood01	ASTAR531-07
			Calolydella Wood03	ASTAR519-07
			Calolydella Wood04	ASTAR507-07
			Calolydella Wood01	ASTAR495-07
			Calolydella Wood01	ASTAR484-07
			Nemorilla Wood01	ASTAR473-07
			Eucelatoria aurea	ASTAR556-07
			Eucelatoria aurea	ASTAR544-07
			Eucelatoria aurea	ASTAR532-07
			Calolydella Wood05	ASTAR520-07
			Calolydella Wood04	ASTAR508-07
			Calolydella Wood01	ASTAR496-07
			Calolydella Wood01	ASTAR485-07
			Nemorilla Wood02	ASTAR474-07
			Eucelatoria aurea	ASTAR557-07
			Eucelatoria aurea	ASTAR545-07
			Eucelatoria aurea	ASTAR533-07
			Calolydella Wood06	ASTAR521-07
			Calolydella Wood04	ASTAR509-07
			Calolydella Wood01	ASTAR497-07

			Calolydella Wood01	ASTAR486-07
			Nemorilla Wood02	ASTAR475-07
			Eucelatoria aurea	ASTAR558-07
			Eucelatoria aurea	ASTAR546-07
			Eucelatoria aurea	ASTAR534-07
			Calolydella Wood04	ASTAR522-07
			Calolydella Wood04	ASTAR510-07
			Calolydella Wood02	ASTAR498-07
			Calolydella Wood01	ASTAR487-07
			Actinodoria argentifrons	ASTAR476-07
			Eucelatoria Wood07	ASTAR559-07
			Eucelatoria aurea	ASTAR547-07
			Eucelatoria aurea	ASTAR535-07
			Calolydella Wood04	ASTAR523-07
			Calolydella Wood04	ASTAR511-07
			Calolydella Wood02	ASTAR499-07
			Calolydella Wood01	ASTAR488-07
			Actinodoria Wood01	ASTAR477-07
			Eucelatoria Wood09	ASTAR560-07
			Eucelatoria aurea	ASTAR548-07
			Eucelatoria aurea	ASTAR536-07
			Eucelatoria aurea	ASTAR524-07
			Calolydella Wood04	ASTAR512-07
			Calolydella Wood02	ASTAR500-07
			Calolydella Wood01	ASTAR489-07
			Calolydella Wood01	ASTAR478-07
			Eucelatoria Wood08	ASTAR561-07
			Eucelatoria aurea	ASTAR549-07
			Eucelatoria aurea	ASTAR537-07
			Eucelatoria aureaDHJ01	ASTAR525-07
			Calolydella Wood04	ASTAR513-07
			Calolydella Wood02	ASTAR501-07
			Calolydella Wood01	ASTAR490-07
			Calolydella Wood01	ASTAR479-07
			Eucelatoria Wood13	ASTAR562-07
			Eucelatoria aurea	ASTAR550-07
			Eucelatoria aurea	ASTAR538-07
			Eucelatoria aureaDHJ01	ASTAR526-07
			Calolydella Wood04	ASTAR514-07
			Calolydella Wood02	ASTAR502-07
			Calolydella Wood01	ASTAR491-07
			Calolydella Wood01	ASTAR480-07
			Eucelatoria Wood11	ASTAR563-07
			Eucelatoria aurea	ASTAR551-07
			Eucelatoria aurea	ASTAR539-07
			Eucelatoria Wood01	ASTAR527-07
			Calolydella Wood04	ASTAR515-07
			Calolydella Wood02	ASTAR503-07
			Calolydella Wood01	ASTAR492-07
			Calolydella Wood01	ASTAR481-07
			Eucelatoria Wood11	ASTAR564-07
			Eucelatoria aurea	ASTAR552-07

			Eucelatoria aurea	ASTAR540-07
			Eucelatoria armigeraDHJ05	ASTAR528-07
			Calolydella Wood04	ASTAR516-07
			Calolydella Wood04	ASTAR504-07
	Nematodes	22	Dracunculus insignis	RFNPM001-07
			Dracunculus insignis	RFNPM002-07
			Dracunculus insignis	RFNPM003-07
			Dracunculus insignis	RFNPM004-07
			Dracunculus insignis	RFNPM005-07
			Dracunculus insignis	RFNPM006-07
			Dracunculus insignis	RFNPM007-07
			Dracunculus insignis	RFNPM008-07
			Dracunculus insignis	RFNPM009-07
			Dracunculus insignis	RFNPM010-07
			Dracunculus insignis	RFNPM011-07
			Dracunculus lutrae	RFNPM083-07
			Dracunculus lutrae	RFNPM084-07
			Dracunculus lutrae	RFNPM085-07
			Dracunculus lutrae	RFNPM086-07
			Dracunculus lutrae	RFNPM087-07
			Dracunculus lutrae	RFNPM088-07
			Dracunculus lutrae	RFNPM089-07
			Dracunculus lutrae	RFNPM090-07
			Dracunculus lutrae	RFNPM091-07
			Dracunculus lutrae	RFNPM092-07
			Dracunculus lutrae	RFNPM093-07
	Aphids and adelgids	12	Aphis sedi	RFBAC211-07
			Schizolachnus obscurus	RFBAC212-07
			Eulachnus rileyi	RFBAC213-07
			Pemphigus spyrothecae	RFBAC214-07
			Aphis fabae	RFBAC215-07
			Macrosiphum	RFBAC216-07
			Aphis	RFBAC217-07
			Eulachnus rileyi	RFBAC218-07
			Schizolachnus pineti	RFBAC219-07
			Eulachnus rileyi	RFBAC220-07
			Schizolachnus obscurus	RFBAC221-07
			Macrosiphum	RFBAC222-07
	Scale insects	12	Dynaspidiotus californicus	LHASA695-07
			Dynaspidiotus californicus	LHASA696-07
			Coccidae	LHASA691-07
			Coccidae	LHASA692-07
			Chionaspis pinifoliae	LHASA697-07
			Chionaspis pinifoliae	LHASA698-07
			Stramenaspis kellogi	LHASA704-07
			Stramenaspis kellogi	LHASA705-07
			Stramenaspis kellogi	LHASA706-07
			Coccidae	LHASA699-07
			Coccidae	LHASA700-07
			Carlusapis	LHASA701-07
	Bark beetles	12	Xyleborinus alni	LHMD117-07
			Xyleborinus alni	LHMD118-07

			Xyleborinus alni	LHMD119-07
			Xyleborinus alni	LHMD120-07
			Xyleborinus alni	LHMD121-07
			Xyleborinus alni	LHMD122-07
			Xyleborinus alni	LHMD123-07
			Xyleborinus alni	LHMD124-07
			Hylurgops	LHMD125-07
			Hylurgops	LHMD126-07
			Hylurgops	LHMD127-07
			Hylurgops	LHMD128-07
	Grasshoppers	12	Psylla	RDBAB822-07
			Psylla	RDBAB823-07
			Psylla sanguinea	RDBAB824-07
			Psyllinae	RDBAB825-07
			Psyllinae	RDBAB826-07
			Psyllinae	RDBAB827-07
			Psyllinae	RDBAB828-07
			Psyllinae	RDBAB829-07
			Psylla	RDBAB830-07
			Neophyllura arctostaphyli	RDBAB831-07
			Trioza chenopodii	RDBAB832-07
			Aphalara	RDBAB833-07
	Various insects	24	Cercopidae	RDBAB834-07
			Cercopidae	RDBAB835-07
			Cercopidae	RDBAB836-07
			Cercopidae	RDBAB837-07
			Membracidae	RDBAB838-07
			Membracidae	RDBAB839-07
			Membracidae	RDBAB840-07
			Kleidocerys franciscanus	RDBAB841-07
			Phlegyas annulicrus	RDBAB842-07
			Phlegyas annulicrus	RDBAB843-07
			Cymus luridus	RDBAB844-07
			Kleidocerys franciscanus	RDBAB845-07
			Melanoplus dawsoni	RFOR023-06
			Melanoplus fasciatus	RFOR024-06
			Melanoplus femurrubrum	RFOR025-06
			Melanoplus gladstoni Scudder	RFOR026-06
			Melanoplus infantilis	RFOR027-06
			Melanoplus packardii	RFOR028-06
			Melanoplus sanguinipes	RFOR029-06
			Phoetaliotes nebrascensis	RFOR030-06
			Aeropedellus clavatus	RFOR031-06
			Ageneotettix deorum	RFOR032-06
			Melanoplus dawsoni	RFOR033-06
			Melanoplus fasciatus	RFOR034-06
	Lepidoptera	188	Nisoniades godma	MHAHG784-06
			Ouleus dilla baru	MHAHG753-06
			Quadrus contubernalis	MHAHG795-06
			Quasimellana antipazina	MHAHG789-06
			Staphylus vulgata	MHAHH001-06
			Lento xanthina	MHAHH067-06

			Astraptes MYST	MHAHH180-06
			Astraptes NUMT	MHAHH170-06
			Gonioterma anna	MHAYA823-06
			Eulepte concordalis	MHMXD532-06
			Microthyris alvinalis	MHMXD546-06
			Omiodes confusalis	MHMXD556-06
			Omiodes humeralis	MHMXD549-06
			Pantographa expansalis	MHMXD550-06
			Agaraea minuta	MHMXC524-06
			Antiblemma amarga	MHMXC567-06
			Coenipeta bibitrix	MHMXC547-06
			Cosmosoma hercyna	MHMXC532-06
			Glaucostola guttipalpis	MHMXC526-06
			Melese monima	MHMXC529-06
			Oraesia nobilis	MHMXC539-06
			Pachydota saduca	MHMXC537-06
			Archaeoprepona meander	MHMXC659-06
			Callicore lyca	MHMXC645-06
			Callicore pacifica	MHMXC644-06
			Chlosyne hippodrome	MHMXC647-06
			Egchiretes nomimus	MHMXC602-06
			Eueides aliphera	MHMXC646-06
			Eurema albula	MHMXC634-06
			Ganyra josephina	MHMXC642-06
			Hemiceras conspirata	MHMXC615-06
			Hemiceras nigrescens	MHMXC616-06
			Memphis pithyusa	MHMXC654-06
			Mimoides branchus	MHMXC628-06
			Munona iridescens	MHMXC621-06
			Opsiphanes cassina	MHMXC658-06
			Opsiphanes invirae	MHMXC657-06
			Opsiphanes quiteria	MHMXC660-06
			Phoebis philea	MHMXC639-06
			Phoebis sennae	MHMXC638-06
			Phyprosopus parthenope	MHMXC572-06
			Pterourus birchalli	MHMXC629-06
			Pyrisitia nise	MHMXC635-06
			Smyrna blomfildia	MHMXC655-06
			Ancyluris inca	MHMXC716-06
			Anteros formosus	MHMXC722-06
			Cacostatia sapphira	MHMXC738-06
			Caligo atreus	MHMXC673-06
			Calydna sturnula	MHMXC721-06
			Cosmosoma zurcheri	MHMXC758-06
			Dysschema jansonis	MHMXC739-06
			Emesis brimo	MHMXC703-06
			Euselasia chrysippe	MHMXC710-06
			Euselasia hygenius	MHMXC699-06
			Juditha caucana	MHMXC727-06
			Lophocampa modesta	MHMXC746-06
			Mesosemia asa	MHMXC718-06
			Mesosemia carissima	MHMXC719-06

			Nymphidium onaeum	MHMXC726-06
			Pachydota drucei	MHMXC743-06
			Panthiades bitias	MHMXC675-06
			Pelochyta misera	MHMXC745-06
			Pseudolycaena damo	MHMXC688-06
			Accinctapubes albifasciata	MHMXC822-06
			Amycles anthracina	MHMXC784-06
			Chloropaschia granitalis	MHMXC801-06
			Cosmosoma auge	MHMXC761-06
			Cosmosoma teuthras	MHMXC763-06
			Dasyvesica lophotalis	MHMXC815-06
			Deuterollyta chlorisalis	MHMXC809-06
			Eucereon aeolum	MHMXC770-06
			Eucereon atrigutta	MHMXC772-06
			Eucereon latifascia	MHMXC771-06
			Halysidota grandis	MHMXC781-06
			Isanthrene echemon	MHMXC764-06
			Lactura subfervens	MHMXC793-06
			Lampruna rosea	MHMXC782-06
			Macrocneme cabimensis	MHMXC766-06
			Quadraforma obliqualis	MHMXC806-06
			Chloropaschia mennusalis	MHCPA001-06
			Omphalocera cariosa	MHCPA057-06
			Paridnea holophaealis	MHCPA065-06
			Pococera sadotha	MHCPA004-06
			Macalla niveorufa	MHCPA165-06
			Carthara abrupta	MHMXD288-06
			Anacrusis nephrodes	MHMXD167-06
			Euglyphis braganzoides	MHMXD181-06
			Gippius sumptuosus	MHMXD157-06
			Hyblaea puera	MHMXD126-06
			Monoloxis flavicintalis	MHMXD127-06
			Acraga coa	MHMXD222-06
			Asellodes daphnites	MHMXD235-06
			Divana diva	MHMXD228-06
			Erastria decrepitaria	MHMXD232-06
			Opisthoxia cluana	MHAGB796-07
			Ptychamalia cumana	MHAGB743-07
			Anisodes melitia	MHAGB873-07
			Epimescis conjugaria	MHAGB819-07
			Ischnopteris bryifera	MHAGB834-07
			Oxydia sociata	MHAGB875-07
			Pseudasellodes fenestraria	MHAGB881-07
			Sicya medangula	MHAGB898-07
			Ergavia carinenta	MHMXG053-07
			Erosia veninotata	MHMXG065-07
			Holochroa ochra	MHMXG004-07
			Leuciris fimbriaria	MHMXG038-07
			Macaria abydata	MHMXG060-07
			Macaria gambarina	MHMXG017-07
			Opisthoxia asopis	MHMXG022-07
			Opisthoxia uncinata	MHMXG021-07

			Aleuron chloroptera	MHMXG168-07
			Schidax squamaria	MHMXG133-07
			Athyrma adjutrix	MHMXG253-07
			Bahaia empirica	MHMXG244-07
			Dasylophia basitincta	MHMXG246-07
			Lirimiris auriflua	MHMXG250-07
			Malocampa hibrida	MHMXG245-07
			Mimallo amalia	MHMXG234-07
			Nystalea morona	MHMXG255-07
			Othorene purpurascens	MHMXG230-07
			Paramacna nattereri	MHMXG237-07
			Othorene verana	MHMXG223-07
			Ptiloscola dargei	MHMXG209-07
			Polythrix kanshul	MHAHJ499-07
			Pythonides amaryllis	MHAHJ466-07
			Salatis canalis	MHAHJ519-07
			Oxynthes corusca	MHAHJ552-07
			Polyctor enops	MHAHJ549-07
			Cobalus virbius	MHAHJ704-07
			Staphylus vulgata	MHAHJ696-07
			Nascus paulliniae	MHAHJ676-07
			Perichares perichares	MHAHJ655-07
			Anastrus sempiternus	MHAHJ752-07
			Astraptes anaphus	MHAHJ790-07
			Calliades zeutus	MHAHJ785-07
			Heliopetes alana	MHAHJ761-07
			Quadrus francesius	MHAHJ754-07
			Cabares potrillo	MHAHJ833-07
			Camptopleura theramenes	MHAHJ872-07
			Phanes aletes	MHAHJ856-07
			Quasimellana servilius	MHAHJ840-07
			Euselasia eubule	MHAIA653-07
			Lyropteryx lyra	MHAIA615-07
			Mesosemia coelestis	MHAIA655-07
			Mesosemia grandis	MHAIA607-07
			Parelbella macleannani	MHAHJ910-07
			Ascia monuste	MHMXG492-07
			Chlosyne gaudialis	MHMXG534-07
			Cissia tiessa	MHMXG548-07
			Dismorphia amphiona	MHMXG516-07
			Dynamine hoppi	MHMXG547-07
			Heraclides anchisiades	MHMXG481-07
			Memphis cleomestra	MHMXG556-07
			Memphis niedhoeferi	MHMXG550-07
			Napeogenes tolosa	MHMXG564-07
			Parides lycimenes	MHMXG482-07
			Caligo eurilochus	MHMXG635-07
			Eueides lybia olympia	MHMXG625-07
			Eueides procula	MHMXG595-07
			Morpho granadensis	MHMXG646-07
			Dalcerides mesoa	MHMXG686-07
			Minacraga plata	MHMXG678-07

			Podalia contigua	MHMXG740-07
			Prolimacodes badia	MHMXG695-07
			Druentia inscita	MHMXG760-07
			Euglyphis lepta	MHMXG806-07
			Euglyphis scaptia	MHMXG800-07
			Macrosoma bahiata	MHMXG771-07
			Rolepa innotabilis	MHMXG839-07
			Conchylodes arcifera	MHMXG929-07
			Ephoria lybia	MHMXG852-07
			Hoterodes ausonia	MHMXG908-07
			Stenoma patens	MHMXG887-07
			Stenoma patens	MHMXG888-07
			Stenoma patens	MHMXG893-07
			Leucerodes terminalis	MHMXH079-07
			Cimicodes purpurea	MHMXH240-07
			Simopteryx torquataria	MHMXH221-07
			Simopteryx torquataria	MHMXH222-07
			Eusarca nemora	MHMXH249-07
			Eutomopepla artena	MHMXH252-07
			Hygrochroma olivinaria	MHMXH230-07
			Macrotes commatica	MHMXH195-07
			Paragonia tasima	MHMXH248-07
			Pyrinia sanitaria	MHMXH241-07
			Amaxia apyga	MHMXH357-07
			Argyroeides notha	MHMXH338-07
		1566	Carathis byblis	MHMXH367-07
