# Supplementary material for: A universal DNA mini-barcode for biodiversity analysis
Source: BMC Genomics. 2008 May 12;9:214. doi: 10.1186/1471-2164-9-214 (PMC2396642; doi:10.1186/1471-2164-9-214)
Supplement: Additional file 2 — Mini-barcodes of 130 bp can be PCR amplified from the majority of specimens using a single universal primer set for all major eukaryotic lineages. [file 1471-2164-9-214-S2.pdf]

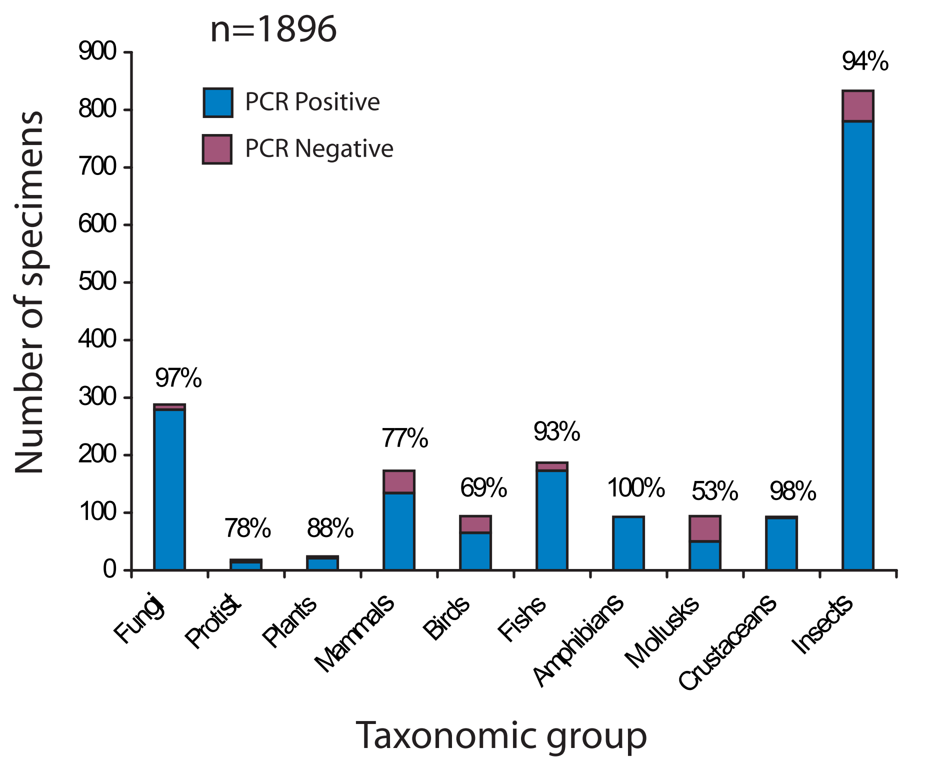
n=1896
PCR Positive
PCR Negative
Number of specimens
900
800
700
600
500
400
300
200
100
0
97%
78%
88%
77%
69%
93%
100%
53%
98%
94%
Fungi
Protist
Plants
Mammals
Birds
Fishs
Amphibians
Mollusks
Crustaceans
Insects
Taxonomic group
